# Studies on the Expression of Sesquiterpene Synthases Using Promoter-β-Glucuronidase Fusions in Transgenic *Artemisia annua* L

**DOI:** 10.1371/journal.pone.0080643

**Published:** 2013-11-22

**Authors:** Hongzhen Wang, Junli Han, Selvaraju Kanagarajan, Anneli Lundgren, Peter E. Brodelius

**Affiliations:** Department of Chemistry and Biomedicine, Linnaeus University, Kalmar, Sweden; Nazarbayev University, Kazakhstan

## Abstract

In order to better understand the influence of sesquiterpene synthases on artemisinin yield in *Artemisia annua*, the expression of some sesquiterpene synthases has been studied using transgenic plants expressing promoter-GUS fusions. The cloned promoter sequences were 923, 1182 and 1510 bp for β-caryophyllene (CPS), *epi*-cedrol (ECS) and β-farnesene (FS) synthase, respectively. Prediction of *cis*-acting regulatory elements showed that the promoters are involved in complex regulation of expression. Transgenic *A. annua* plants carrying promoter-GUS fusions were studied to elucidate the expression pattern of the three sesquiterpene synthases and compared to the previously studied promoter of amorpha-4,11-diene synthase (ADS), a key enzyme of artemisinin biosynthesis. The CPS and ECS promoters were active in T-shaped trichomes of leaves and stems, basal bracts of flower buds and also in some florets cells but not in glandular secretory trichome while FS promoter activity was only observed in leaf cells and trichomes of transgenic shoots. ADS, CPS, ECS and FS transcripts were induced by wounding in a time depended manner. The four sesquiterpene synthases may be involved in responsiveness of *A. annua* to herbivory. Methyl jasmonate treatment triggered activation of the promoters of all four sesquiterpene synthases in a time depended manner. Southern blot result showed that the *GUS* gene was inserted into genomic DNA of transgenic lines as a single copy or two copies. The relative amounts of CPS and ECS as well as germacrene A synthase (GAS) transcripts are much lower than that of ADS transcript. Consequently, down-regulation of the expression of the *CPS*, *ECS* or *GAS* gene may not improve artemsinin yield. However, blocking the expression of *FS* may have effects on artemisinin production.

## Introduction

Secondary metabolites released from aerial organs into air and roots into soil play important roles in plant responsiveness to various external signals, such as direct and indirect defenses, plant reproduction and plant-plant interactions. Many of these secondary products are terpenoids. The most common emitted terpenoids are mono- and sesquiterpenes. The constituents of such emissions differ for different varieties and even in different tissues of the same plant [1]–[6].

Many terpene synthases involved in the biosynthesis of the emitted terpenoids have been cloned and characterized. For instance, β-caryophyllene synthase (CPS) and (3R)-linalool synthase (LIS) were found to be involved in responsiveness to wounding and fungal elicitors in *Artemisia annua* [1], [2]. (*E*)-β-CPS is suggested to be involved in indirect defenses of maize and rice, even though it is not expressed in all varieties of maize [7], [8]. (*E*)-β-Farnesene synthase (FS), which can be induced by herbivore in some maize genotypes, was cloned and characterized from maize [9]. This enzyme has been cloned from a number of plants, such as *A. annua* [10], Norway spruce [11], peppermint [12], and *Citrus junos* [13]

Many terpene synthases catalyze the formation of a single product while some terpene synthases are involved in the formation of product mixtures. In maize, such an enzyme (terpene synthase I) catalyzes the formation of (*E*)-farnesene, (*E*)-nerolidol, and (*E*,*E*)-farnesol after herbivore attack [9]. Two sesquiterpene synthases encoded by two florally expressed genes catalyze the biosynthesis of almost all sesquiterpenes found in floral volatile blends from *Arabidopsis* [14].

Artemisinin is an endoperoxide sesquiterpene lactone of high value isolated from the Chinese herbal medicinal plant *A. annua*. Artemisinin Combination Therapies (ACTs) are and continue to be effective against multi-resistant malaria parasites and are at present the preferred drug to fight this disease [15]. The amounts of artemisinin in the plants are, however, small and range from 0.01% to 0.8% on a dry weight basis between different varieties of the plant [16]. The amount of artemisinin was increased by establishing hybrid plants and up to 1.4% has been obtained [17] and recently a yield of around 2% of DW was reported [18]. At present, the production of artemisinin does not meet the demand of the drug. It is important to find effective methods to improve artemisinin production in the plant in order to reduce the price of ACTs.

The biosynthesis of artemisinin is fairly well understood as far as the enzymology is concerned. Cyclization of farnesyl diphosphate (FDP) to amorpha-4-11-diene by amorph-4,11-diene synthase (ADS) is the initial step of the artemisinin biosynthetic pathway [19] and amorph-4,11-diene is the committed precursor [20]. This first committed step appears to be rate limiting for artemisinin biosynthesis. In the following step, amorpha-4,11-diene is hydroxylated to yield artemisinic alcohol. This reaction is catalyzed by a cytochrome P450 dependent amorpha-4,11-diene 12-hydroxylase (CYP71AV1) [21]. This enzyme can covert the amorpha-4,11-diene all the way to artemisinic acid, which is the final enzymatic intermediate and precursor of arteannuin B [22]. Alternatively, the artemisinic alcohol can also be oxidized to the corresponding aldehyde by alcohol dehydrogenase 1 (ADH1) [23]. In the next step, the aldehyde is reduced to dihydroartemisinic aldehyde by artemisinic aldehyde Δ11(13) reductase (DBR2) [24]. The final enzymatic step in artemisinin biosynthesis is catalyzed by aldehyde dehydrogenase 1 (ALDH1) [25], which converts the aldehyde to the corresponding acid. The intermediates artemisinic acid and dihydroartemisinic acid are converted to arteannuin B and artemisinin, respectively, in a non-enzymatic reaction. All the genes encoding these enzymes have been cloned and the recombinant enzymes at least partly characterized. Two chemotypes of *A. annua* have been characterized. One is high in artemisinic acid and arteannuin B (low artemisinin producer; LAP) and the other is high in dihydroartemisinic acid and artemisinin (high artemisinin producer; HAP) [22], [26]. Glandular trichome specific expression of biosynthetic genes [21], [27], [28], [29] shows that artemisinin is sequestered and localized to glandular secretory trichomes (GSTs) of *A. annua* [30], [31].

**Figure 1:**
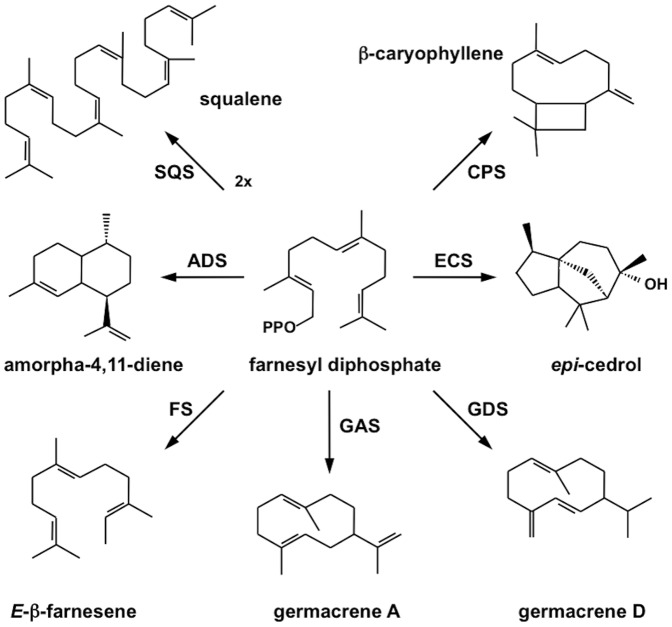
Enzymes in *Artemisia annua* utilizing farnesyl diphosphate as substrate. ADS: amorpha-4,11-diene synthase; CPS: β-caryophyllene synthase; ECS: *epi*-cedrol synthase; FS: β-farnesene synthase; GAS: germacrene A synthase; GDS: germacrene D synthase; SQS: squalene synthase; PPO: diphosphate moiety

The first and rate-limiting enzyme of artemisinin biosynthesis is ADS converting FDP to amorpha-4,11-diene. A number of other enzymes using FDP as substrate are present in *A. annua* and may reduce the yield of artemisinin due to a competition for the substrate FDP (Figure 1). These include, in addition to ADS [19], the sesquiterpene synthases β-CPS [2], *epi*-cedrol synthase (ECS) [32], germacrene A synthase (GAS) [33], and FS [11]. In addition, squalene synthase (SQS) utilizing FDP to produce squalene may compete for the substrate [34]. The possible influence of the activity of these enzymes on artemisinin yield has been shown in a few studies. Using the antisense technique, the activities of SQS [35] and β-CPS [36] were down-regulated in transgenic *A. annua* plants leading to improved yields of artemisinin. Furthermore, over-expression of the endogenous farnesyl diphosphate synthase (FDS) [37] or hydroxy-methyl-glutaryl-CoA reductase (HMGR) [38] resulted in increased artemisinin yields; most likely due to increased availability of FDP for ADS.

The activity of various sesquiterpene synthases is reflected in the composition of extracts and essential oils from *A. annua,* which varies between different cultivars. In most cultivars of *A. annua,* germacrene D, β-caryophyllene and β-farnesene are main constituents [3], [4], [6], [39]–[42]. The presence of a number of other sesquiterpenes of other structural types, such as α-copaene, α-humulene, α-selinene and spathulenol, indicate that some additional sesquiterpene synthases are expressed in *A. annua*.

Some studies on the expression of sesquiterpene synthases in *A. annua* have been reported. The relative expression of ADS, β-CPS, and ECS transcripts in different tissues were studied by qPCR to determine the relative expression of these enzymes [5]. The expression of ADS is very high in flower buds and young leaves, while ECS is highly expressed in old leaves. The expression of β-CPS is considerable higher in young leaves and flower buds than in old leaves. Furthermore, this enzyme is induced in response to wounding or fungal elicitors indicating that β-caryophyllene plays a role in plant defense [1].

We have previously reported on studies on the ADS promoter using a promoter-GUS fusion in transgenic *A. annua* [27]. The recombinant ADS promoter was specifically active in GSTs, which is the site of artemisinin biosynthesis. In order to better understand the influence of other sesquiterpene synthases on artemisinin yield, we have extended these studies and cloned the promoters of the *CPS*, *ECS* and *FS* genes. These three genes were randomly selected from the sesquiterpene synthase that have been cloned from *A. annua*. An additional sesquiterpene synthase (germacrene A synthase) has been cloned from *A. annua* but this enzyme was not included in the promoter studies. The cloned promoters have been fused to the GUS reporter gene and transgenic *A. annua* plants expressing these fusions have been established. Here we report on studies on the activity of these promoters in different tissues as reported by GUS staining. The response of the wild-type promoters to wounding and methyl jasmonate treatment have been studied by qPCR.

## Materials and Methods

### Plant materials

Seeds of *A. annua* var. Chongqing were obtained from the Southwest University in Chongqing China. The Chongqing cultivar is a high artemisinin producer.

Plants of *A. annua* were grown under 16 h light and 8 h dark at 22 °C to a height of approximately 1 m followed by flower buds induction at 8 h light and 16 h dark at 22 °C. Flower buds, young leaves, old leaves, stems and roots were collected for GUS staining. Samples of these tissues were also frozen in liquid nitrogen and ground to a fine powder for RNA extraction.

### Promoter cloning

Genomic DNA was extracted from fresh young leaves of *A. annua* using the CTAB method [43]. The GenomeWalker Universal Kit (Clontech) was used to amplify the promoter region of the *CPS*, *ECS* and *FS* genes by PCR using primers listed in Table 1 as instructed by the manufacturer. Two adaptor primers [44] and two gene specific primers (primers 1–6; Table 1) of each gene were used in the PCR reactions. The nucleotide sequence obtained was used to design primers for PCR amplification (Table 1) of 923, 1182 and 1510 bp fragments of the CPS, ECS and FS promoters, respectively, using Pfu polymerase (Fermentas). The primers used (primers 7–12; Table 1) carried *Eco*RI and *Nco*I restriction site for cloning of the fragments into the modified plant transformation vector pCAMBIA1381Z.

**Table 1:** Nucleotide sequence of primers used. Restriction sites are underlined; F  =  forward; R  =  reverse.

Primer no	Name	Application	Primer Sequence
1	CPS-GS1	GenomeWalker	5′-ATCGAAGATGAGAAACTGGTCTGCCC-3′
2	CPS-GS2	GenomeWalker	5′-GGAGGAAAGTGGACAATGGGCCGAAT-3’
3	ECS-GS1	GenomeWalker	5′-CATAGGCAAGAAACTGATCTCCCCAAAT-3’
4	ECS-GS2	GenomeWalker	5′-CAGAAGGAAAATTTGCATTGGGGCGTA-3’
5	FS-GS1	GenomeWalker	5′-TGGCGGATAACATCTGGTTTCGTGCTA-3’
6	FS-GS2	GenomeWalker	5′-ACCAAAGGTGACGTAGATGAAGAGAATGA-3’
7	CPS_F_	Cloning	5′-GGAATTCATCGGCGTGCGGTGCATGCCTGTA-3′
8	CPS_R_	Cloning	5′-CATGCCATGGAGAAGTTTGGATCTATGTAT-3’
9	ECS_F_	Cloning	5′-CCGGAATTCATCCATAAATTTATTGTT-3’
10	ECS_R_	Cloning	5′-CATGCCATGGCTTGATAAAAGATGCGAATT-3’
11	FS_F_	Cloning	5′-GGAATTCATCACTCAAGGATGGATGTATT-3’
12	FS_R_	Cloning	5′-CATGCCATGGCTCAAAATCTTGCGAGTTTGA-3’
13	FS_F_	qPCR	5′-ACCTATGGCTTGATGATAGCGAGAT-3’
14	FS_R_	qPCR	5′-GCACCACTTTCCTTAGAATAGCATTC-3’
15	NPTII_F_	probe	5′-TGGGCACAACAGACAATCGGCTGC-3′
16	NPTII_R_	probe	5′-TGCGAATCGGGAGCGGCGATACCG-3′

### Transformation vector construction

The pCAMBIA1381Z vector (CambiaLabs) carrying the GUS-gene was used for the transformation of *A. annua*. This vector had previously been modified in that the plant selection gene was changed from hygromycin to kanamycin [27].

The cloned promoter regions were double-digested with *Nco*I/*Eco*RI and separately inserted into the modified pCAMBIA1381Z vector digested with the same restriction enzymes. The plant transformation vectors obtained (pCAMBIA1381Z::*pCPS::GUS,* pCAMBIA1381Z::*pECS::GUS* and pCAMBIA1381Z::*pFS::GUS*) were introduced into *E. coli* BL21, which was grown on LB medium containing kanamycin (50 mg/l). pCAMBIA1381Z*::GUS* lacking a promoter was used as control. The vectors were purified using the GeneJet Plasmid Miniprep Kit (Fermentas) and the plant transformation vectors were introduced into *A. tumefaciens* EHA105 by the freeze and thaw method. *A. tumefaciens* EHA105 carrying the plant transformation vectors were grown on YEP medium containing kanamycin (100 mg/L) and rifampicin (40 mg/L) at 28°C to an OD_600_  =  0.8–1. The cells were collected by centrifugation and resuspended in MSMO liquid medium to an OD_600_  =  0.3–0.5.

### Plant transformation

Seed sterilization and germination as well as plant transformation was carried out as previously described [27].

### GUS assay

Leaf primordia at the apex and expanded leaves at different nodes, stems, roots from shoots and flowering plants, and flower buds were sampled from transgenic *A. annua* plants to perform GUS analysis. GUS histochemical staining of the various plant tissues was carried out as previously described [45]. GUS stained tissues were studied under a microscope (Nikon ECLIPSE E400) and photographs were taken using a digital camera (Nikon DP11).

### qPCR

Different tissues of transgenic *A. annua* plants were sampled to analyze expression pattern by qPCR using β-actin as reference gene. RNA was extracted using Purelink™ Plant RNA Reagent (Invitrogen) according to the manufacture’s instruction. Genomic DNA was removed by treatment with DNase I (Fermentas). RNA (1 µg) was reverse transcribed using RevertAid™ H Minus-MuLV reverse transcriptase (Fermentas) primed with 0.5 µg oligo(dT)_18_ primer. RNA was removed from the cDNA obtained by treatment with RNase H (Fermentas).

The qPCR was performed on a 7500 qPCR thermocycler (Applied Biosystems) using primers for β-actin, ADS, CPS, ECS, GUS [6], [27], [44] or FS (primers 13 and 14 in Table 1). First single-stranded cDNA was used as template in a 20 µL reaction mixture containing 10 µL Power SYBR® Green PCR Master Mix (Applied Biosystems) and 2 pmol of each primer. qPCR thermal cycling was performed at 50°C (2 min), 95°C (10min), 40 cycles at 95°C (15 sec), 60°C (1 min) and finally a dissociation state at 95°C (15 sec), 60°C (1 min) and 95°C (15 sec). Triplet samples were run for each cDNA sample.

### Wounding and hormonal treatments

Propagation of transgenic lines was carried out by shoot cuttings. Plants with similar morphology were selected 2 months after roots had appeared and were used for wounding and hormone experiments.

Leaves of transgenic of *A. annua* plants were wounded at vegetative or reproductive stage by cutting (3–5 mm) along the midrib with a razor blade. Wound responsiveness was checked by GUS staining. Three transgenic lines were tested and three plants of the same variety transformed with the control vector were used as control. The response of the wild-type promoters to wounding was determined by qPCR using non-transgenic plants.

Transgenic plants were sprayed with 300 µM methyl jasmonate (MeJA) + 0.01% Tween 20. Each plant was sprayed with 80 mL MeJA solution from 4 directions to ensure treatment of the entire plant. Three control plants of the same transgenic line were sprayed with 0.01% Tween 20. Treated plants were kept in open air for 3 h until there was no liquid on leaf surfaces. Subsequently, the plants were returned to standard growth conditions for 69 h. Middle leaves were sampled for expression analysis by GUS-staining. For each variety, three lines were checked. For each line, three technical replicates were carried out. The response of the wild-type promoters to MeJA was determined by qPCR using non-transgenic plants.

### Southern blot

Genomic DNA was digested completely by *Eco*RI or *Hind*III, and eletrophoretically separated on an agarose gel (0,7% w/v), then transferred to a positively charged nylon membrane (Boheringer Mannheim GmbH). The 686 bp *NPTII* probe was labeled with digoxigenin by PCR using primers 15 and 16 (Table 1). Hybridization was carried out overnight at 42°C. Disodium 3-(4-methoxyspiro {1,2-dioxetane-3,2'-(5'-chloro) tricyclo 3.3.1.1^3,7^] decan}-4-yl) phenyl phosphate (CSPD) (Roche Molecular Biochemicals) was used as substrate for chemiluminescent detection, after high stringency washes. The membrane was exposed to X-ray film for 30-60 min to obtain the desired image strength.

### Statistic methods

Grubbs test (G  =  |Suspect value-x_mean_ | /s) was used to test for outliers. The outliers were rejected if G_calculated_ > G_critical_ at P  =  0.05. The critical value of G is 1.155 when the sample size is three [46].

## Results and Discussion

### Analysis of promoter nucleotide sequences

The nucleotide sequences of the promoter regions upstream to the start codon were cloned by the genome walking method. The transcription start site (TSS) (labeled +1) of the cloned promoters was predicted using the TSSP software (http://linux1.soft-berry.com/berry.phtml) as summarized in Table 2. Using the PLACE (http://www.dna.affrc.go.jp/PLACE/) and PlantCARE (http://bioinformatics.psb.ugent.be/webtools/plantcare/html/) databases, we localized putative TATA- and CAAT-boxes of the recombinant promoters as summarized in Table 2.

**Table 2:** Some features of the cloned sesquiterpene promoters.

promoter	Size	*Putative	**Putative	TATA-box	**Putative	CAAT-box	DataBank
	(bp)	TSS	TATA box	sequence	CAAT-box	sequence	entry
ADS	1929	51	–29 to –24	TATAAA	–74 to –70	CAATT	JX870081
CPS	920	46	–29 to –24	TATAAA	–54 to –50	CAATT	FJ870127
ECS	1119	49	–34 to –27	TATATATA	–77 to –73	CAAAT	FJ870129
FS	1507	25	–31 to –26	TATATA	–71 to –68	CAATT	JX870082

* The position of the TSS (+1) is given as bases upstream of the ATG start codon.

** The position of the TATA- and CAAT-boxes are given relative to the TSS.

Putative *cis*-acting regulatory elements of the three cloned promoters were predicted using the PlantCARE and PLACE software as summarized in Table 3 and shown in the Figures S1-S5. Here the ADS promoter, previously cloned and characterized [27], has been included. The promoters of the four *A. annua* sesquiterpene synthases carry some putative *cis*-acting regulatory elements in common but also some for each promoter unique elements. It is obvious that the four sesquiterpene synthase promoters are differently regulated and that they all carry a large number of putative *cis*-acting regulatory elements.

**Table 3:** Number of putative *cis*-acting regulatory elements in sesquiterpene synthase promoters from *Artemisia annua*.

*Cis*-acting	Sequence	Promoter				Description
element		ADS	CPS	ECS	FS	
3-AF3 binding site	**CACTATCTAAC**			1		part of a conserved DNA module array (CMA3)
ABRE	**ACGTGG(T/C) TACGTG**		1	2		*cis*-acting element involved in the abscisic acid responsiveness
ACE	**AAACCGGTTA ACGTGGA**	1	1	1	1	*cis*-acting element involved in light responsiveness
ACTTTA motif	**ACTTTA**	3	1		1	*cis*-acting regulatory element involved in auxin induction of oncogenes
AE-box	**AGAAACAA**	1			-	part of a module for light response
AG-motif	**AGATCCAA**		1			*cis*-acting regulatory element that confers responsiveness to wounding and elicitors
ARE	**TGGTTT**	2			3	*cis*-acting regulatory element essential for the anaerobic induction
as-2-box	**GATAatGATG**		1			involved in shoot-specific expression and light responsiveness
ATCT-motif	**AATCTAATCT**			1		part of a conserved DNA module involved in light responsiveness
AuxRR core	**GGTCCAT**	1				*cis*-acting regulatory element involved in auxin responsiveness
box 4	**ATTAAT**	1		5	3	part of conserved DNA module involved in light responsiveness
box 1	**TTTCAAA**	2	1	1	4	light responsive element
Box-W1	**TTGACC**	2				fungal elicitor responsive element
CAT-box	**GCCACT**			1		*cis*-acting regulatory element related to meristem expression
CBFHV	**(A/G)(C/T)CGAC**	1				binding site of dehydration-responsive element (DRE) binding proteins (DREBs)
CGCG-box	**(A/C/G)CGCG(G/T/C)**	1				CGCG-box found in promoters of many genes under Ca++/calmodulin regulation
CGTCA-motif	**CGTCA**		1	1		*cis*-acting regulatory element involved in the MeJA-responsiveness
chs-CMA1a	**TTACTTAA**				1	part of a light responsive element
Circadian	**CAANNNNATC**	2				*cis*-acting regulatory element involved in circadian control
E-box	**CAnnTG**	10	7	3	1	MYC recognition site conferring abscisic acid, dehydration, cold and freezing induction
ERE	**ATTTCAAA**	1				ethylene-responsive element
Eri-box3	**AAACCAATT**		1			elicitor responsive element
	**CACGTT**					
G-box	**CAGGTC**	3	5	3	3	*cis*-acting regulatory element involved in light responsiveness
	**CACATGG**					
GA-motif	**AAGGAAGA AAACAGA**	1			1	part of light responsive element
GAG-motif	**AGAGAGT GGAGATG**				3	part of light responsive element
GARE-motif	**TCTGTTG AAACAGA**	1			1	gibberellin-responsive element
GATA-motif	**AAGATAAGATT**			2		part of a light responsive element
GCN4-motif	**TGTGTCA TGAGTCA**	2			1	*cis*-acting element involved in endosperm expression
GT1-motif	**GGTTAA AATCCACA**	1	1	1	2	light responsive element
HSE	**AAAAAATTTC**	2		1	3	*cis*-acting element involved in heat stress responsiveness
L-box	**AAATTAACCAAC**				1	part of light responsive element
MBS	**TAACTG**	2	2		1	MYB binding site involved in drought-inducibility
MNF1	**GTGCCC(A/T)(A/T)**			1		light responsive element
MRE	**AACCTAA**			1		MYB binding site involved in light responsiveness
O2-site	**GATGACATGA**			1		*cis*-acting regulatory element involved in zein metabolism regulation
P-box	**CCTTTTG**				1	gibberellin-responsive element
Py-rich strech	**TTTCTTCTCT**	1				*cis*-acting element conferring high transciption levels
RAA-motif	**CAACA**	3	2	1	4	*cis*-acting element recognized by AP2/ERF transcription factors
RY-element	**CATGCATG**				1	*cis*-acting element involved in seed-specific regulation
Skn-1-motif	**GTCAT**	6	2	2	3	*cis*-acting regulatory element required for endosperm expression
Sp1	**CC(C/A)CCC**	1				light responsive element
	**GTTTTCTTAC**					
TC-rich repeat	**ATTTTCTTCA**	1		1	3	*cis*-acting element involved in defense and stress responsiveness
	**ATTCTCAAC**					
TCA-element	**GAGAAGAATA**	1				*cis*-acting element involved in salicylic acid responsiveness
TCT-motif	**TCTTAC**	1			2	part of light responsive element
TGA-element	**AACGAC**	1	1			auxin-responsive element
TGACG-motif	**TGACG**		1	1		*cis*-acting regulatory element involved in the MeJA-responsiveness
T/G-box	**AACGTG**	1	1		1	*cis*-acting regulatory element involved in the MeJA-responsiveness
WUN motif	**AATTTCC**	9	5	9	6	wound-responsive element

**Figure 2:**
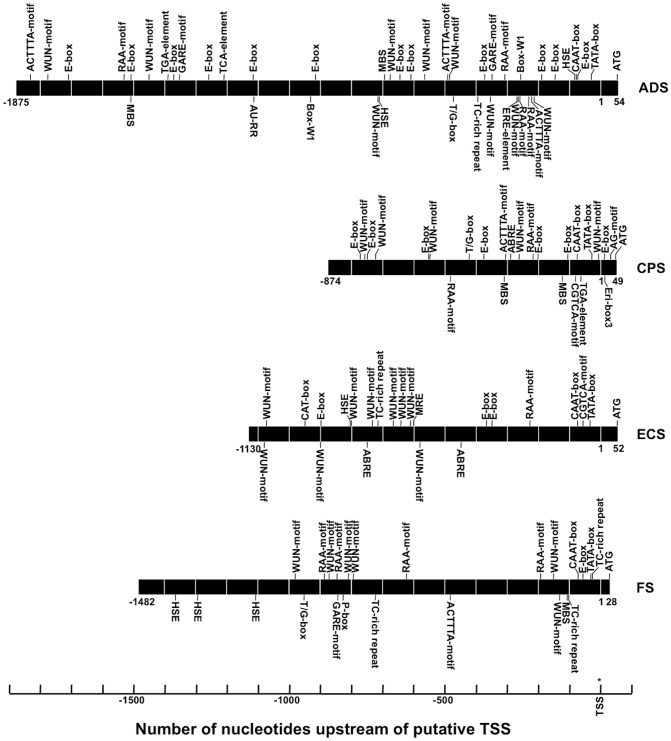
Position of putative *cis*-acting regulatory elements known to be involved in responsiveness towards external factors in the four cloned sesquiterpene synthase promoters. Elements marked above the promoters are located to the (+)-strain and the elements marked under the promoters are located to the (–)-strain.

In plants, sesquiterpene synthase genes are involved in the biosynthesis of communication and defense related terpenoids. Defense terpenoids may be induced by insects, pathogens, wounding or MeJA. We will restrict our discussion of putative *cis*-acting elements of the cloned sesquiterpene synthase promoters to those known to be involved in responsiveness towards external factors, such as wounding, hormones, abiotic and biotic elicitors. The four recombinant sesquiterpene synthase promoters carry a number of putative *cis*-acting regulatory elements involved in responsiveness to various external factors (Figure 2 and Table 3). There are 514 putative transcription factors (TFs) classified in 48 families listed in the Plant Transcription Factor Database (http://planttfdb.cbi.edu.cn/index.php?sp= Aan) for *A. annua*. Some of these TFs are involved in the regulation of the promoters studied.

In *Arabidopsis thaliana,* there are six signal-responsive genes (*AtSR1* to *6*), which are related to the tobacco ethylene-responsive gene *NtER1* encoding a calmodulin-binding protein [47]. The *AtSR* genes are rapidly and differentially induced by environmental signals such as extreme temperatures, UV-B, salt, and wounding, hormones such as ethylene and abscisic acid (ABA), and signal molecules such as MeJA, H_2_O_2_, and salicylic acid (SA) [47]. AtSR1 targets the nucleus and specifically recognizes a 6-bp CGCG-box (A/C/G)CGCG(G/T/C). One or more CGCG *cis*-acting elements are found in promoters of genes involved in ethylene, ABA and SA signaling, and light signal perception. One putative CGCG-box was found in the ADS and ECS promoters (Table 3).

Two MeJA-responsive *APETALA2*/ ethylene response factor TFs (AP2/ERF), *i.e.* AaERF1 and AaERF2, have recently been cloned from *A. annua* [48]. The AP2/ERF TFs bind to CBF2 ([G/a][T/c]CGAC) and RAA (CAACA) motifs. One CBF2 motif was found in the ADS, CPS and FS promoters, while the ADS, CPS, ECS and FS promoters carried three, two, one and four RAA motifs, respectively (Table 3).

The effects of the AaERF1 and AaERF2 have been studied on the ADS promoter [48]. Transient co-expression of *AaERF2* and a *pADS*::*GUS* construct in tobacco resulted in a three-fold increased activity of the ADS promoter. Over-expression of AaERF1 or AaERF2 in transgenic *A. annua* plants resulted in elevated transcript levels of ADS (2- to 8-fold) and in an increased accumulation of artemisinin [49] These results demonstrate that AaERF1 and AaERF2 are two positive regulators of the ADS promoter and possibly also of other promoters.

Basic/helix-loop-helix (bHLH) TFs may be involved in the JA responsiveness of plants [49]. There are 35 such proteins listed in the *A. annua* TF database. Most bHLH TFs bind to the E-box sequence (CANNTG), which also is recognized by MYB and bZIP TFs. The number of E-boxes found in the studied promoters varied; ten, seven, three and one for the ADS, CPS, ECS and FS promoters, respectively (Table 3). These E-boxes may be involved in the response of *A. annua* to JA.

In addition to the JA-responsive motifs discussed above, we have also found some other JA-responsive *cis*-acting regulatory elements in the sesquiterpene synthase promoters. The CGTCA motif, involved in the MeJA-responsive gene expression in barley (*Hordeum vulgare*) [50], was found in the CPS (one copy) and ECS (one copy) promoters and one T/G-box (AACGTG) each in the ADS, CPS and FS promoters (Table 3). The T/G box binds the MYC TF in tomato and *Arabidopsis* involved in JA signaling [51].

Thus, the four sesquiterpene synthase promoters carry a number of JA responsive *cis*-acting elements, which may be involved in the response of *A annua* plants to MeJA. In fact, treatment of plants of *A. annua* with MeJA results in the induction of ADS and other enzymes of artemisinin biosynthesis such as FDS, CYP71AV1, DBR2 and ALDH1 leading to increased production of artemisinin [28], [52], [53].

The ABA-responsive element (ABRE) is a major *cis*-acting element (PyACGTGGC) in ABA-responsive gene expression. One and three such ABA-responsive elements (TACGTG) were found in the CPS and ECS promoters, respectively (Table 3). A single copy of the ABRE is not sufficient to mediate ABA regulation unless a coupling *cis*-element such as coupling element 1 (CE1) (TGCCACCGG) [54], coupling element 3 (CE3) (ACGCGTGTCCTC) [55] or dehydration responsive element (DRE) (TACCGACAT) [56] is present. We have not found any of these coupling elements in the CPS promoter indicating that the single ABRE element of this promoter does not mediate ABA regulation. However, a response to ABA can be seen when the promoter carries multiple copies of the ABRE element [57] and therefore we may expect a response to ABA of the ECS promoter, which carries two ABRE elements (Table 3). It is well established that bHLH TFs are involved in the response of plants to ABA [58]. As discussed above these proteins bind to the E-box sequence and all four sesquiterpene synthase promoters carry such sequences (Table 3). The ABRE and E-box *cis*-acting elements may both be involved in the response of the promoters studied to ABA.

Treatment of *A. annua* plants with ABA (10 µM) resulted in the induction of a number of genes encoding enzymes involved in artemisinin biosynthesis [59]. The expression levels of HMGR, FDS, CYP71AV1 and cytochrome P450 reductase (CPR) were significantly induced while ADS only showed a slight increase.

The TCA-element (GAGAAGAATA) is involved in responsiveness of genes to SA [60]. This element was first characterized as a *cis*-acting element involved in salicylic acid responsiveness and systemic acquired resistance in wild cabbage (*Brassica oleracea*) [61]. Only the ADS promoter carries one such element with the sequence GAGAAGAAAA. Treatment of *A. annua* plants with 1 mM SA resulted in a temporary peak in the expression of the *ADS* gene [62].

A number of auxin-responsive elements have been found in the ADS, CPS and FS promoters. The TGA-element (AACGAC) is found in the ADS (one copy) and CPS (one copy) promoters. The ACTTTA motif is essential for tissue-specific and auxin-regulated expression of the *rolB* oncogene in plants [63]. Such motifs were found in the ADS (three copies), CPS (one copy) and FS (one copy) promoters. Finally, one AU-RR core sequence (GGTCCAT), which is involved in auxin responsiveness, was found in the ADS promoter.

Furthermore, the putative gibberellin (GA)-responsive element (the GARE-motif AAACAGA) is present in the ADS and FS promoters (one copy each). In addition, the FS promoter carries one copy of the (GA)-responsive pyrimidine box (P-box; CCTTTTG); a conserved element in the upstream sequence of GA inducible genes in cereal seeds [64]. Treatment of *A. annua* with gibberellic acid 3 (GA_3_) results in an increased formation of artemisinin, indicating an increased expression of the key regulatory enzyme ADS [24], [65]–[67]. In one study, it was shown by qPCR that the level of ADS transcripts was increased around three times after treatment with 20 mg/L GA_3_ by soil drenching [67].

Only the ADS promoter contains the putative ethylene responsiveness ERE-element (ATTTCAAA) (one copy). The TC-rich repeat (ATTTTCTCCA) was previously described in tobacco (*Nicotiana tabacum*) as a *cis*-acting element involved in defense and stress responsiveness [68]. TC-rich repeats are found in the promoters of ADS (one copy), ECS (one copy) and FS (three copies).

The WRKY TFs constitute a large family of TFs in plants [69]. They function via interaction with other proteins such as MAP kinases, 14-3-3 proteins or calmodulin and they bind to box-W1 elements ([C/T]TGAC[C/T]). WRKY TFs may be involved in the simultaneous regulation of different processes. In cotton, the GaWRKY1 is regulating the activity of cadinene synthase 1 (CAD1) involved in the biosynthesis of sesquiterpene phytoalexins [70]. The CAD1 promoter carries two box-W1 motifs to which the GaWRKY1 may bind. For *A. annua,* there are 29 WRKYs listed in the plant transcription factor database. One of these (AaWRKY1) has been cloned [71].

The fungal elicitor responsive box-W1 element, which interacts in *Arabidopsis* with members of the WRKY transcription factor family to mediate responses to wounding or pathogen responses [72], is only found in the ADS promoter in two copies (Table 3). Transient expression of the *A. annua* transcription factor AaWRKY in agroinfiltrated leaves of *A. annua* resulted in increased levels of ADS (7x) and other biosynthetic enzymes, such as HMGR (5x), CYP71AV1 (3x) and DBR2 (4x) indicating that several of the genes encoding enzymes involved in artemisinin biosynthesis are co-induced by binding of AaWRKY to W-boxes [72]. In fact, the CYP71AV1 [44] and DBR2 [K. Yang *et al*., unpublished] promoters also carry two box-W1 motifs each. A recent study showed that foliar application of an elicitor (chitosan) resulted in increase of dihydroartemisinic acid and artemisinin by 72% and 53%, respectively, in *A. annua* [73]. Furthermore, semi-quantitative RT-PCR showed increased levels of ADS, CYP71AV1 and DBR2 transcripts after application of chitosan.

An elicitor preparation from the phytopathogenic fungus *Verticillium dahliae* induces CPS in leaves of *A. annua* [1]. The recombinant CPS promoter does not carry any of the elicitor responsive box-W1 or TC-rich repeat elements. However, an EL1-box3 sequence (AAACCAATT), which is another elicitor responsive element of plant promoters [74], is found in the CPS promoter. In addition, the CPS promoter carries a *cis*-acting regulatory AG-motif (AGATCCAA) that confers responsiveness to wounding and elicitors [75]. The wound-inducible *MYB* gene *NtMYB2* is up-regulated by an AG-motif binding protein (AGP1), which is a GATA-type zinc finger. The other three promoters do not carry any EL1-box3 sequence or AG-motif.

The WUN motif (consensus AATTTCC) is present in the four promoters in a relative large number, *i.e.* 9, 5, 9 and 6 copies (Table 3) in the ADS, CPS, ECS and FS promoters, respectively, indicating that all four sesquiterpene synthase genes may be induced by wounding. A slight induction of ADS and other enzymes involved in artemisinin biosynthesis was observed after wounding of *A. annua* leaves [76]. This induction resulted in a slight increase in artemisinin yield. It was shown that the *CPS* gene is induced by wounding [1]. To our knowledge, no reports on the induction of the *ECS* or *FS* gene by wounding have been published.

In addition, the E-boxes discussed above are also involved in responses to abiotic elicitors. They are MYC recognition site conferring dehydration, cold and freezing induction. The MYB binding site (MBS; TAACTG) is involved in drought-inducibility. In *Arabidopsis*, MBS sequences are the binding site for the proteins AtMYB1 and AtMYB2 involved in regulation of genes that are responsive to water stress [77] and in *Petunia hybrida* for MYB.Ph3 involved in regulation of flavonoid biosynthesis [78]. MBS sequences were found in the ADS (two copies), CPS (two copies) and FS (one copy) promoters. The ADS, ECS and FS promoters carry two, one and three copies, respectively, of the HSE *cis*-acting element (AAAAAATTTC) involved in heat stress responsiveness.

Lulu *et al*. [79] showed that chilling affects the transcript levels of ADS and CYP71AV1 in *A annua* cultures. Using qPCR, they observed after 24 h 11- and 7-fold increases in ADS and CYP, respectively, in cultures chilled to 4°C for 30 min compared to non-chilled cultures. Higher temperatures (42°C) did not seem to affect the transcript levels of either ADS or CYP71AV1. Yang *et al*. [80] also showed marked increases in ADS in response to chilling. It was also shown that a significant increase in ADS occurred in response to a 1-h dose of UV light. Furthermore, when plant roots were allowed to dry for 6 h, an increased of ADS in shoots was observed [80]. In contrast, water logging decreased ADS.

### Southern blot

**Figure 3:**
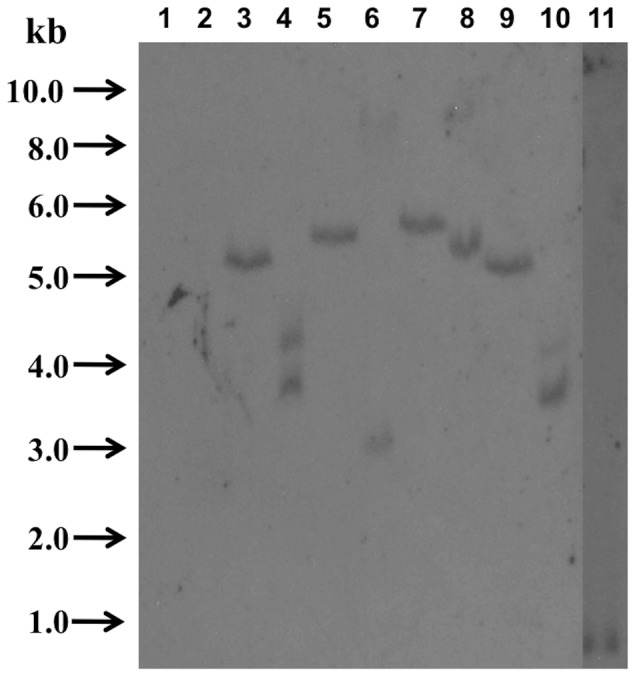
Southern blot of genomic DNA isolated from wild-type (lane 1-2) and transgenic (lane 3-10) plants using a digoxigenin-labeled NPTII probe. Lane 1: wild-type plant digested with *Hind*III; lane 2: wild-type plant digested with *Eco*RI; lane 3: transgenic plant carrying the *pECS-GUS* fusion digested with *Hind*III: lane 4: transgenic plant carrying the *pECS-GUS* fusion digested with *Eco*RI; lane 5: transgenic plant carrying the *pFS-GUS* fusion digested with *Hind*III: lane 6: transgenic plant carrying the *pFS-GUS* fusion digested with *Eco*RI; lane 7: transgenic plant carrying the *pCPS-GUS* fusion digested with *Hind*III: lane 8: transgenic plant carrying the *pCPS-GUS* fusion digested with *Eco*RI; lane 9: transgenic plant carrying the *pADS-GUS* fusion digested with *Hind*III: lane 10: transgenic plant carrying the *pFS-GUS* fusion digested with *Eco*RI; lane 11: positive control (882 bp fragment carrying the *NPTII* gene). Sample size: 10–15 µg/lane.

In order to determine the transgene copy number, the various transgenic plants were analyzed by Southern blotting. Genomic DNA from wild-type and transgenic plants was digested completely by *Eco*RI or *Hind*III, and the fragments obtained were analyzed by Southern blotting using a 686 bp hybridization probe binding to the *NPTII* gene (Figure 3). All lines showed positive hybridization bands. The transgenes were integrated into the genome with a single copy (the *pCPS::GUS* and *pFS::GUS* constructs) or two copies (the *pECS::GUS* and *pADS::GUS* constructs) (Figure 3).

### Activity of the CPS Promoter

In order to study the temporal and spatial expression of the *CPS* gene, transgenic *A. annua* plants carrying the *pCPS::GUS* construct were produced using *A. tumefaciens.* Twenty-three positive T_0_ transgenic lines were selected by PCR (primers listed in Table 1). Fifteen plants transformed with pCAMBIA1381Z*::GUS* (lacking a promoter) were obtained and five randomly selected lines were used as controls.

**Figure 4:**
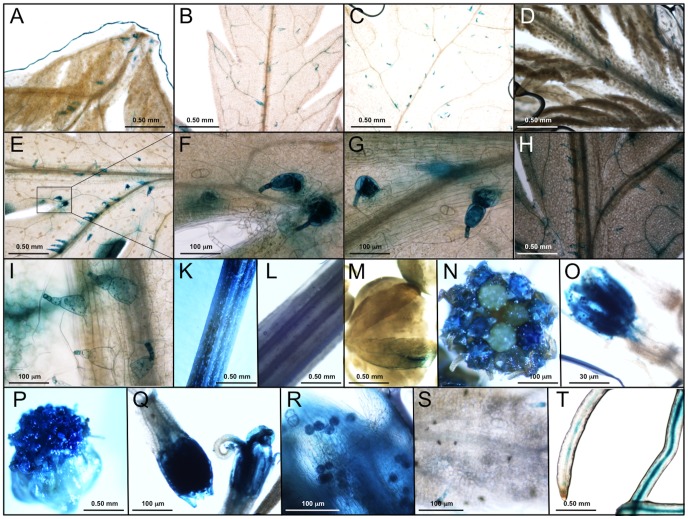
GUS expression controlled by the *CPS* promoter in transgenic plants of *Artemisia annua*. A: leaf primordia; B: lower leaf; C: leaf at bottom at early vegetative stage; D: leaf primordia; E: leaf at upper node; F: close-up of panel E; G: leaf at upper node; H: leaf at lower node at late vegetative stage; I: leaf at lower node at late vegetative stage; K: stem; L: stem; M: flower buds; N: flowers at early flowering stage; O: floret; P: flowers at late flowering stage; Q: florets; R: pollen; S: flower bracts; T: roots.

Tissues of primary transgenic lines were tested for CPS promoter activity by histochemical GUS activity staining. The same expression pattern was observed for all transgenic lines but the intensity of the staining varied. For further studies, we selected the five lines showing the strongest staining. GUS expression controlled by the CPS promoter was exclusively located to T-shaped trichomes (TSTs) of leaf primordia and expanded leaves (Figures 4A to 4I). No GUS staining was observed in GSTs (Figures 4F, 4G), which is in contrast to the ADS promoter, which exhibits specific expression in GSTs where artemisinin is biosynthesized. Leaf veins of some old leaves showed GUS staining (Figures 4B, 4H). On lower/older leaves of transgenic plants at early vegetative stage, a larger fraction of the TSTs showed GUS expression (Figures 4A to 4C). There is no obvious difference in GUS expression between upper and lower leaves of older plants (Figures 4D, 4E, 4H). However, mature leaves of older seedlings showed lower GUS expression. It appeared that GUS expression of TSTs gradually became stronger to a high level during TST development and that the expression of GUS varied during TST development. TSTs on the midrib or the adaxial surface showed stronger GUS staining than those on the abaxial surface. The GUS expression in TSTs on midrib near leaf petiole is stronger than that on/near leaf veins close to the leaf tips (Figures 4A to 4H). This indicates that TSTs at different locations on the same leaf surface are at different developmental stages, which may implicate that CPS expression is different at different locations on the same leaf. GUS expression in stem is located to TSTs and the site where it was sampled by cutting (Figures 4K, 4L), which indicates that CPS is wound inducible (This is discusses further below). Basal bracts of flower buds showed GUS staining (Figure 4M). Corolla, pollen of hermaphroditic florets near to the marginal pastille florets of flowers at early stage of development also exhibited GUS expression (Figures 4N to 4O). Corolla, style and stigma of flowers at late stage of development showed GUS expression in two kinds of florets (Figures 4P to 4R). These results indicate that CPS expression is different at different stages of flower development and that the highest level of expression is seen at the full blooming stage. This is in agreement with the finding that the level of β-caryophyllene was highest at the full blooming stage [4], [6]. Only a few TSTs of flowers showed GUS staining (Figure 4S). Finally, GUS staining was observed in vascular tissues of root (Figure 4T).

Except for the expression in roots, the results obtained are in good agreement with those of Cai *et al.* [1] showing that the *CPS* gene is expressed in all tissues of *A. annua* except in roots. In this study, the CPS promoter is specifically active in TSTs of leaves, stem, basal bract of flower buds and some florets cells but not in GSTs.

### Activity of the ECS Promoter

Transgenic *A. annua* plants carrying the *pECS::GUS* construct were produced using *A. tumefaciens* as described above for the CPS promoter. Twenty-six positive T_0_ transgenic lines were selected by GUS staining and PCR (primers listed in Table 1). Fifteen plants transformed with the pCAMBIA1381Z*::GUS* (lacking a promoter) construct were obtained and five randomly selected lines were used as controls.

**Figure 5:**
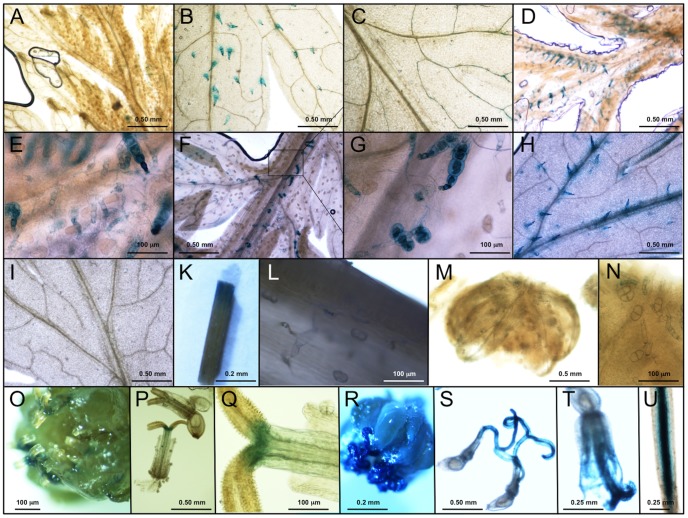
GUS expression controlled by the *ECS* promoter in transgenic plants of *Artemisia annua*. A: leaf primordia; B: lower leaf; C: leaf at bottom at early vegetative stage; D: leaf primordia; E: leaf primordia; F: leaf at upper node; G: close-up of panel F; H: leaf at lower node; I: leaf at bottom at late vegetative stage; K: stem; L: stem ; M: flower buds; N: flower buds; O: flower at early flower stage; P: florets; Q: florets; R: flower at late flower stage; S: hermaphroditic floret; T: pistillate floret; U: root.

All the lines showed similar GUS expression pattern but the intensity of the staining varied. We selected the five lines exhibiting the strongest staining for further experiments. Tissues of transgenic *A. annua* plants at vegetative stage (two stages) and at the reproductive stage were sampled for histochemical GUS staining. At an early vegetative stage, GUS expression was exclusively located to TSTs of leaf primordia and expanded leaves (Figures 5A, 5B). A larger fraction of the TSTs on leaves at lower locations on the plant showed GUS staining comparable to that observed in leaf primordia at apex at an early stage of development (Figures 5A, 5B). Some leaf veins of old leaves at the lowest node showed GUS staining (Figures 5C, 5I). At late vegetative stage, GUS expression is specifically located to TSTs of leaf primordia and young leaves at upper nodes of the plants (Figures 5D, 5H). No GUS-staining was observed in GSTs (Figures 5E to 5G). Midrib, veins and margin of older leaves also showed GUS expression (Figures 5F, 5H). There is no GUS expression in very old leaves at the lowest node of the plant (Figure 5I). We may conclude that ECS expression occurs mainly in TSTs of leaves (Figure 5). Furthermore, TSTs on midrib or adaxial leaf surface and on midrib near leaf petiole showed strong GUS expression (Figures 5B, 5D, 5F, 5H). In conclusion, ECS expression is highly dependent on trichome development. GUS expression in stem was observed in TSTs and at the locus where it was cut during sampling (Figures 5K, 5L) indicating that ECS may be involved in wound-response and plant defense in *A. annua* as discussed below. GUS expression in flower buds is specifically located to TSTs of basal bracts (Figure 5M and 5N). Also in flower buds no staining of GSTs was observed (Figure 5N). Style and stigma of hermaphroditic florets at an early stage of flower development exhibited GUS expression (Figures 5O to 5Q). Stigma and both types of florets were GUS stained at late stages of flower development (Figures 5R to 5T). TSTs on basal bracts also showed GUS staining. GUS staining was observed in vascular tissues of root (Figure 5U). The results showed that ECS is mainly located to TSTs of leaves, stems and flower buds but also in floret cells, but not in GSTs.

### Activity of the FS Promoter

Transgenic *A. annua* plants carrying the *pFS::GUS* construct were produced using *A. tumefaciens* as described above for the CPS promoter. Five positive T_0_ transgenic lines were selected by GUS staining and PCR (primers listed in Table 1). Five plants transformed with the pCAMBIA1381Z*::GUS* (lacking a promoter) construct were used as controls.

**Figure 6:**
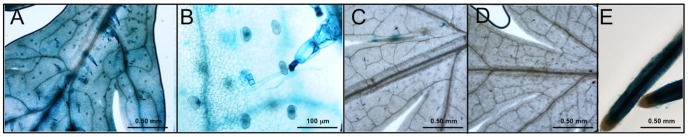
GUS expression controlled by the *FS* promoter in transgenic plants of *Artemisia annua*. A: young leaf; B: close-up of young leaf; C: leaf; D: old leaf; E: root.

All transgenic lines showed similar expression patterns, but different expression levels in individual plants. GUS expression was observed in leaf cells of shoots including GSTs and TSTs (Figures 6A, 6B). FS is the only of the three sesquiterpene synthases studied that shows staining of GSTs (Figure 6B) and therefore most likely is co-expressed with ADS in these cells. At a somewhat later stage of leaf development only a few trichomes were stained (Figure 6C). No GUS staining was observed in old leaves (Figure 6D). Vascular tissues of root showed strong GUS staining (Figure 6E). The GUS expression observed showed a temporal expression in young leaves of shoots.

### Promoter activity in different tissues

The activity of the wild-type sesquiterpene synthase promoters in different tissues of *A. annua* was determined by measuring the transcript levels of the gene by qPCR. β-Actin was used as reference gene. The relative activity of the wild-type promoters in different tissues was compared with the activity of the β-actin promoter, which was set to 1.0. Relative activities were calculated using the 2**^-^** ^ΔΔC**t**^ method [81]. Software REST 2009 (http://www.REST.de.com) was used to analyze the data.

**Figure 7:**
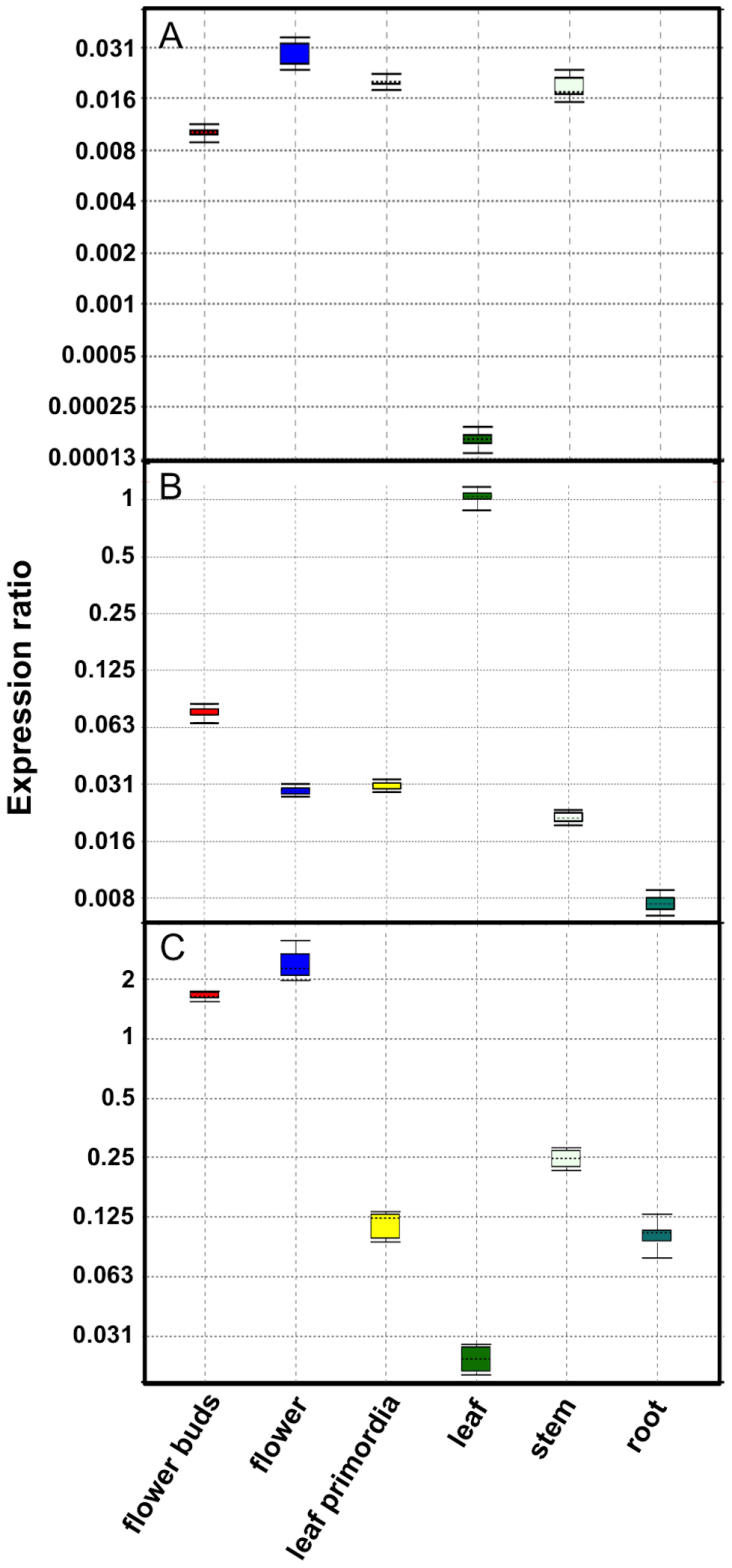
Relative expression of the wild-type *pCPS* (A), *pECS* (B) and *pFS* (C) in different tissues of *Artemisia annua*. All activities are relative to the activity of the β-actin promoter, which was set to 1.0.

The wide-type CPS promoter (*pCPS*) was active in all plant tissues except in roots (Figure 7A), The *pCPS* was highly active in flower buds and leaf primordia but exhibited a relatively low activity in old leaves (Figure 7A). This is consistent with the analysis of CPS transcript levels in *A. annua* plants [5]. Furthermore, the promoter showed highest activity in flowers and the relative activity was ∼200-fold of that in stem (Figure 7A). This result supports the finding that the amount of β-caryophyllene increased sharply at late flower developmental stages [42].

The wild-type ECS promoter (*pECS*) was highly active in flower buds and old leaves while it was moderately activity in flowers and leaf primordia (Figure 7B). The relative activity of *pECS* in old leaves was ∼ 30 times higher than that in leaf primordia (Figure 7B). Very low promoter activity was observed in roots. We have previously reported that the wild-type ECS promoter is very active in old leaves [5], which is confirmed in these studies.

Finally, the wide-type FS promoter (*pFS)* was highly active in flowers (Figure 7C). The *pFS* was moderately active in flower buds but exhibited low activity in old leaf and roots (Figure 7C).

No or low activity of the three sesquiterpene synthase promoters were observed in roots when under the control of the wild-type promoters (Figures 7A, 7B, 7C). However, under the control of the recombinant promoters, GUS-staining was observed in vascular tissues of roots of transgenic plants for CPS (Figures 4T), ECS (Figure 5U) and FS (Figure 6E). No staining was observed for the recombinant ADS promoter [27]. Obviously, there is a difference in the regulation of expression of CPS, ECS and FS in wild-type and transgenic plants. A possible explanation for this difference is that the plant uses a silencing mechanism based on miRNA for the downregulation of sesquiterpene synthase expression in trichome-free roots. This mechanism of down-regulation would not be effective for the GUS transcript leading to the high level of GUS transcripts observed in vascular tissues of roots of transgenic plants.

### Wound-response of recombinant sesquiterpene synthase promoters

**Figure 8:**
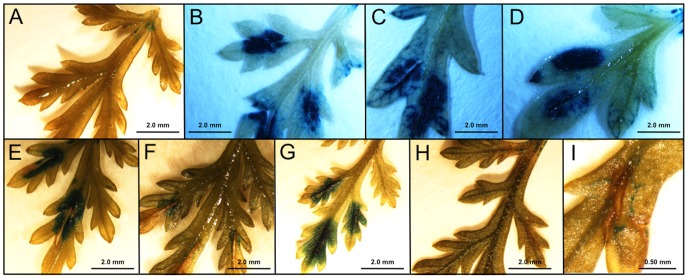
Wounding of leaves of transgenic *Artemisia annua* carrying the *pCPS::GUS* fusion. A: unwounded; B: immediately after wounding; C: 1h; D: 2h; E: 4h; F: 8h; G: 12h; H: 24h; I: 48h.

**Figure 9:**
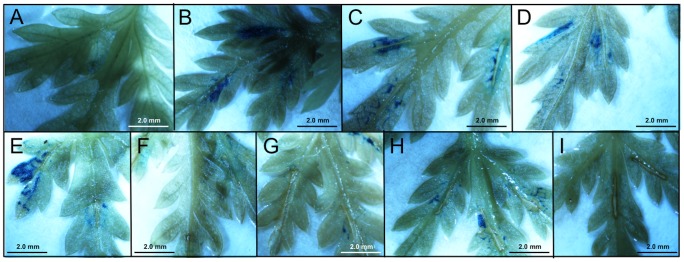
Wounding of leaves of transgenic *Artemisia annua* carrying the *pECS::GUS* fusion. A: unwounded; B: immediately after wounding; C: 1h; D: 2h; E: 4h; F: 8h; G: 12h; H: 24h; I: 48h.

The four promoters studied carry a number of putative *cis*-acting regulatory elements that may be involved in responses of the plant to mechanical wounding (WUN motifs) (Table 3 and Figure 2). In order to investigate the effects of wounding, leaves of transgenic lines at vegetative reproductive stage carrying the sesquiterpene synthases promoter-GUS constructs were wounded by cutting with a razor blade along the midrib (3–5 mm). Subsequently, the wounded leaves were analyzed by GUS staining during a time courses of 48 h after wounding (Figures 8 and 9).

Leaves of transgenic lines carrying the *pCPS::GUS* fusion showed rapid GUS expression at the site of wounding. Strong GUS staining was observed immediately after wounding, *i.e.* around 10 minutes (Figure 8B). A high GUS expression is maintained for at least 12 h (Figures 8C to 8G). At 24 or 48 h, very little or no staining is observed (Figures 8H and 8I).

Wounded leaves of transgenic lines expressing the *pECS::GUS* fusion showed strong GUS staining immediately after wounding (Figure 9B), which was maintained for 4 h. At 8 h after wounding, no staining was observed (Figure 9F). Subsequently, the expression of GUS increased to a visual level at 12 and 24 h (Figures 9G and 9H) and finally to no expression at 48 h (Figure 9I).

Wounded leaves carrying the *pFS::GUS* and *pADS::GUS* constructs did not show any visual GUS expression after wounding (data not shown). However, recently it was shown that ADS is induced by wounding and that the amount of artemisinin is increased around 50% 4 h after wounding [76].

**Figure 10:**
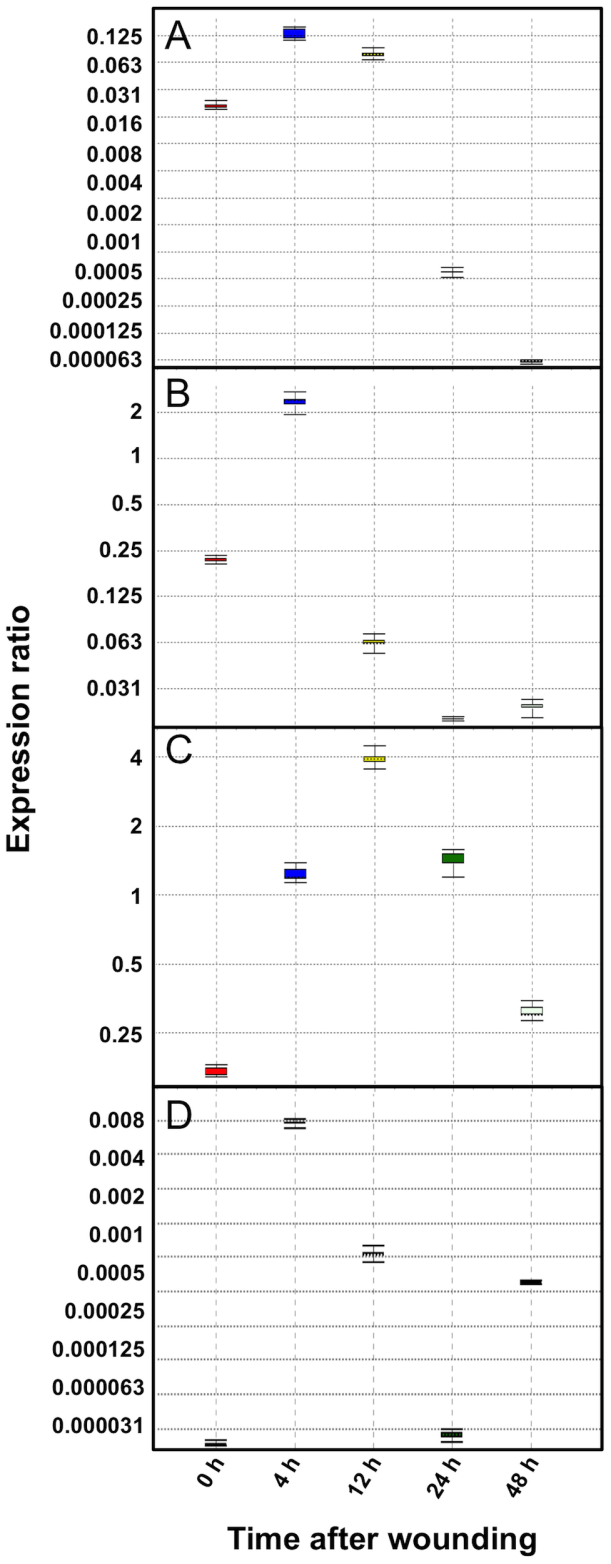
Relative expression of the wild-type *pADS* (A), *pCPS* (B), *pECS* (C), and *pFS* (D) in leaves after wounding. The β-actin promoter activity was set to 1.0.

The activity of the wild-type sesquiterpene synthases promoters in wounded and unwounded leaves of *A. annua* was determined by qPCR. β-Actin was used as reference gene. The relative activity was compared with that of the β-actin promoter, which was set to 1.0 (Figure 10).

The four wild-type sesquiterpene synthase promoters were transiently induced by wounding (Figure 10). A peak in the transcript levels was observed 4 h after wounding for the ADS, CPS and FS promoters, while the ECS promoter showed highest expression at 12h after wounding (Figure 10C). However, from GUS-staining (Figure 9) it is obvious that the ECS promoter is rapidly activated by wounding. The reason for the relatively slow increase in the ECS transcript level observed after wounding may be the comparably high constitutive expression of ECS in leaves [5], which may mask the local wounding response. For the ADS and CPS promoters, the promoter activity decreased at 12 h after wounding. Relatively low but still detectable transcript levels were observed 24 and 48 h after wounding. The ECS promoter activity decreased at 24 and 48 h after wounding but the levels were still higher than that of unwounded leaves. The FS promoter activity decreased at 12h after wounding and exhibited similar level with that of unwounded leaves at 24 and 48 h after wounding.

The fact that an induction of ADS and FS is observed in the qPCR experiments but not in GUS-staining experiments may be explained by the absolute levels of induction. The maximum transcript level of ADS and FS is 0.13- and 0.07-fold, respectively, compared to the level of β-actin transcripts. The sensitivity of the GUS-staining method is not sensitive enough to detect these low levels of induction. On the other hand, the maximum transcript level of CPS and ECS is 2.3- and 3.9-fold higher than that of the β-actin transcript, respectively. Obviously, these high promoter activities are detectable with the GUS-staining technique (Figures 8 and 9).

Our results on the ADS and CPS promoters are consistent with those obtained by semi-quantitative PCR analysis of the transcript levels of ADS [76] and CPS [1] in leaves after mechanical wounding. For ECS and FS, there are no reports on the effects of wounding on the expression of these sesquiterpene synthases. We may conclude that the four sesquiterpene synthase genes are induced by mechanical damage and that the sesquiterpenoids produced by the enzyme may play important roles in the response of *A. annua* to herbivory.

### Response to methyl jasmonate

As discussed above, the four promoters carry a number of putative *cis*-acting regulatory elements involved in the response to MeJA (*e.g.* CBF2, CGTCA-motifs, E-boxes, RAA-motifs and T/G-boxes) (Figure 2; Table 3). To investigate the effects of MeJA on the expression of the four sesquiterpene synthases wild-type and transgenic plants were sprayed with 300 µM MeJA + 0.01% Tween 20. Control plants were sprayed with 0.01% Tween 20. For each experiment three plants were used.

GUS-staining of treated transgenic plants showed that no difference in the expression patterns of GUS could be observed in any of the plants, *i.e*. after MeJA treatment GUS expression was observed in the same tissues as for non-treated plants.

**Figure 11:**
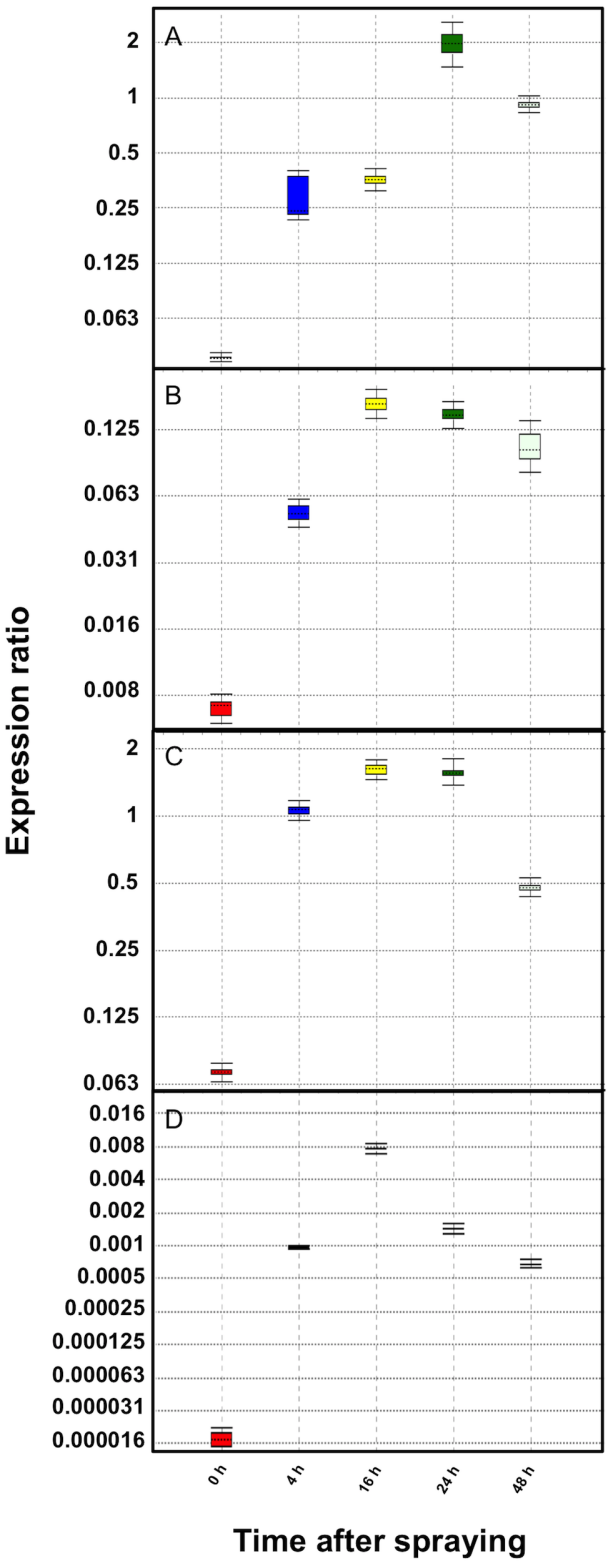
Relative expression of the wild-type *pADS* (A), *pCPS* (B), *pECS* (C) and *pFS* (D) in leaves after spraying MeJA. The β-actin promoter activity was set to 1.0.

Since it is impossible to quantitatively determine the relative levels of GUS expression in transgenic plants by staining, the activity of the wild-type sesquiterpene synthase promoters were determined by qPCR after MeJA-treatment, using β-actin as reference gene. The relative activity was compared with that of β-actin promoter, which was set to 1.0 (Figure 11). The four wild-type sesquiterpene synthase promoters exhibited similar expression pattern after the MeJA-treatment. The increase in transcript levels of CPS, ECS and FS reached a maximum 16 h after the treatment and at this point the transcript level was ∼0.2-, ∼1.6- and ∼0.03-fold the β-actin transcript level, respectively. The ADS promoter activity showed the highest induction 24 h after MeJA-treatment. The activity was ∼2-fold of the β-actin activity. After 48 h all the four sesquiterpene synthases transcript levels decreased to low level but still much higher than that of untreated leaves. In control plants, the transcript levels remained unchanged during the whole time course.

In the case of the *ADS* gene, some studies on the effect of MeJA have been carried out, which are in agreement with the results presented here [28], [52], [53]. The level of ADS transcripts increased during 24 h after treatment with 100 µM MeJA [52]. At this time point, the amounts of dihydroartemisinic acid and artemisinin was increased 1.9- and 1.5-fold, respectively. Furthermore, an indirect evidence of the induction of the *CPS* gene was given as the amount of caryophyllene increased 3-fold after the MeJA treatment [52]. Similarly a 3-fold increase in the amount of germacrene D was reported. Obviously a germacrene D synthase (not yet cloned from *A. annua*) is induced by the MeJA treatment.

In another study, *A. annua* plants of both chemotypes were treated with 100 µM MeJA every second day. After two weeks a 6- and 3-fold increase of ADS transcripts was observed in the HAP and LAP variety, respectively [26]. Other enzymes of artemisinin biosynthesis were also induced by the MeJA treatment. Depending on the chemotype, MeJA treatment resulted in increased amounts of artemisinic acid and arteannuin B in the LAP variety and increased amounts of dihydroartemisinic acid and artemisinin in the HAP variety.

### Relative amounts of sesquiterpene synthase transcripts in A. annua tissues

**Figure 12:**
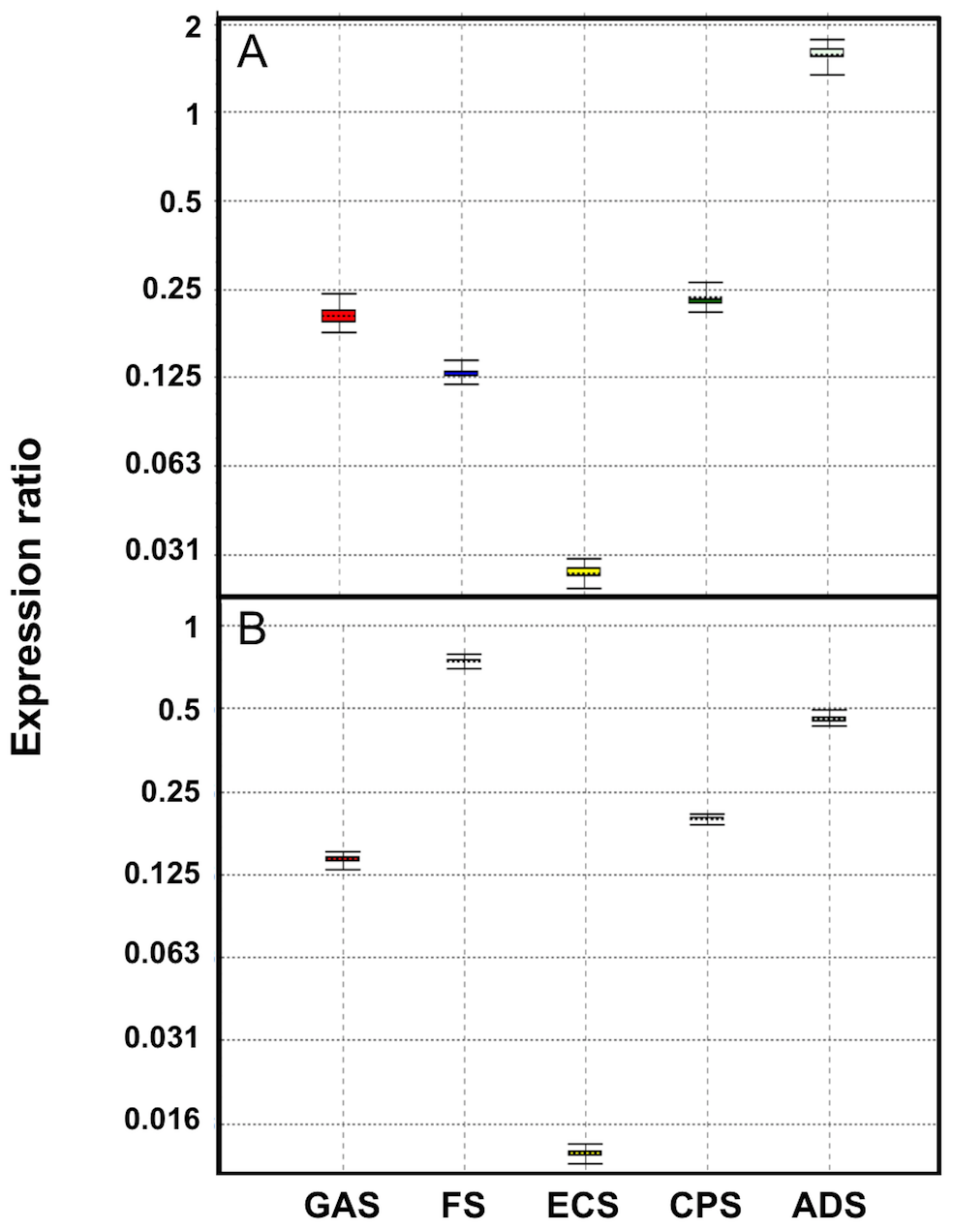
Relative levels of *GAS, FS*, *ECS, CPS* and *ADS* transcripts in flower buds (A) and leaf primordia (B) compared to the β-actin transcript level in *A. annua*. The relative amount of β-actin transcripts was set to 1.0.

In these experiments, we have also included the germacrene A synthase (GAS) using primers as reported previously [5]. The relative amounts of GAS, FS, ECS, CPS and ADS transcripts were compared with that of the β-actin transcript in flower buds and leaf primordia of *A. annua* by qPCR. The relative amount of the β-actin transcript was set to 1.0 (Figure 12). Flower buds and leaf primordial were selected since these tissues carry a relative large number of GSTs, which are the sites of artemisinin biosynthesis. Furthermore, the concentration of artemisinin precusors is high in young tissues [82], [83].

In flower buds, the relative amounts of GAS, FS, ECS, CPS and ADS transcript were 0.14-, 0.74-, 0.01-, 0.20- and 0.46-fold the β-actin transcript level, respectively (Figure 12A). The relative amounts of GAS, ECS and CPS transcript were much lower than that of ADS transcript while the relative amount of FS was slightly higher. In leaf primordia, the relative levels of GAS, FS, ECS, CPS and ADS transcripts were 0.20-, 0.13-, 0.03-, 0.23- and 1.5-fold, the β-actin transcript level, respectively (Figure 12B). All competitive sesquiterpene synthases showed lower transcript levels than ADS. In the Anamed cultivar, relative amounts of GAS, ECS and CPS transcripts were also much lower that of ADS [5].

## Conclusions

The four cloned sesquiterpene synthase promoters show different expression patterns as reported by the histochemical GUS staining. It seems that CPS and ECS do not influence the biosynthesis of artemisinin to any great extent due to their low expression in glandular trichomes (Figures 4 and 5). However, using the antisense technique, the activity of CPS was down-regulated in transgenic *A. annua* plants leading to improved yields of artemisinin [36]. A possible reason for this observation is that the expression of FS, which is expressed in glandular trichomes (Figure 6A and 6B) and competes with ADS for the substrate FDP is down-regulated by the CPS antisense sequence. Unspecific down-regulation of other than the target gene has been shown to occur [84], [85]. The nucleotide sequences of CPS and FS show an identity of 66.4% in the anti-sense target region. Long stretches of identical bases are present in the two sequences, which makes an unspecific down-regulation of FS possible. It would be interesting to investigate the effects of a direct down-regulation of FS on artemisinin yield.

In conclusion, the five sesquiterpene synthases exhibited different expression pattern (Figures 7 and 12). The relative amounts of GAS, ECS and CPS transcripts were much lower than that of ADS transcript in tissues involved in artemisinin biosynthesis, while the relative level of FS transcript was somewhat higher in flower buds. These results are essentially consistent with the results reported for the Anamed cultivar [5]. We may conclude that down-regulation of the *GAS*, *ECS* or *CPS* genes may not improve artemisinin production in *A. annua* to any great extent. However, down-regulation of the *FS* gene may have an effect on the yield of artemisinin.

## Supporting Information

Figure S1 **Colour-code for** ***cis*** **-acting elements present in the four cloned promoters.**
(PDF)

Figure S2 **Nucleotide sequence of the cloned ADS promoter with putative** ***cis*** **-acting elements shown.** Putative TSS is shown in bold. Putative TATA- and CAAT-boxes are underlined.
(PDF)

Figure S3 **Nucleotide sequence of the cloned CPS promoter with putative** ***cis*** **-acting elements shown.** Putative TSS is shown in bold. Putative TATA- and CAAT-boxes are underlined.
(PDF)

Figure S4 **Nucleotide sequence of the cloned ECS promoter with putative** ***cis*** **-acting elements shown.** Putative TSS is shown in bold. Putative TATA- and CAAT-boxes are underlined.
(PDF)

Figure S5 **Nucleotide sequence of the cloned FS promoter with putative** ***cis*** **-acting elements shown.** Putative TSS is shown in bold. Putative TATA- and CAAT-boxes are underlined.
(PDF)
