# Supplementary material for: Studies on the Expression of Sesquiterpene Synthases Using Promoter-β-Glucuronidase Fusions in Transgenic Artemisia annua L
Source: PLoS One. 2013 Nov 22;8(11):e80643. doi: 10.1371/journal.pone.0080643 (PMC3838408; doi:10.1371/journal.pone.0080643)
Supplement: Figure S1 — Colour-code for cis -acting elements present in the four cloned promoters. (PDF) [file pone.0080643.s001.pdf]

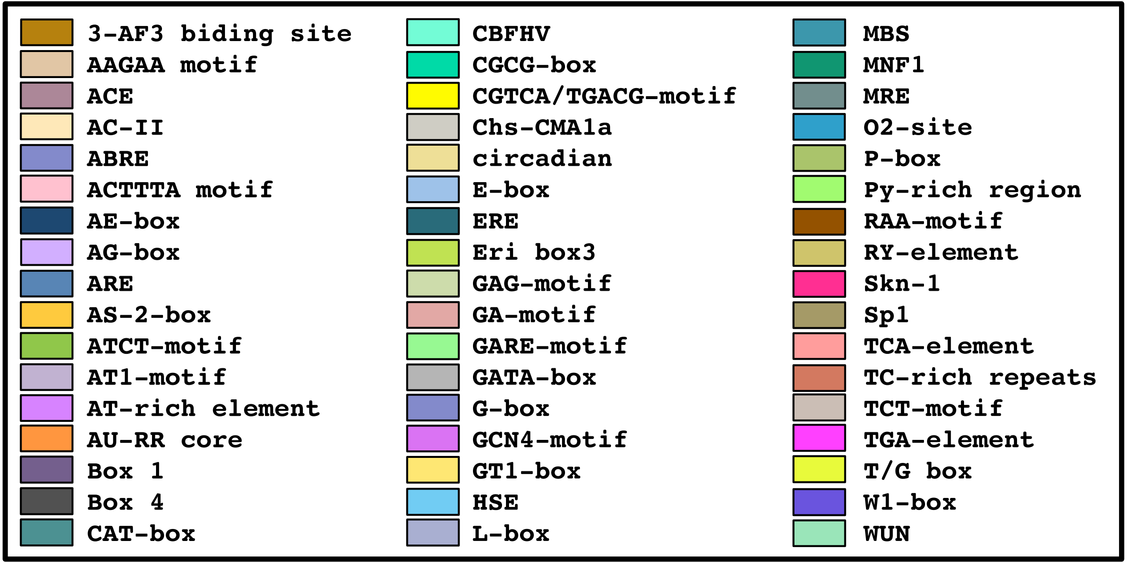
3-AF3 biding site
AAGAA motif
ACE
AC-II
ABRE
ACTTTA motif
AE-box
AG-box
ARE
AS-2-box
ATCT-motif
AT1-motif
AT-rich element
AU-RR core
Box 1
Box 4
CAT-box
CBFHV
CGCG-box
CGTCA/TGACG-motif
Chs-CMA1a
circadian
E-box
ERE
Eri box3
GAG-motif
GA-motif
GARE-motif
GATA-box
G-box
GCN4-motif
GT1-box
HSE
L-box
MBS
MNF1
MRE
O2-site
P-box
Py-rich region
RAA-motif
RY-element
Skn-1
Sp1
TCA-element
TC-rich repeats
TCT-motif
TGA-element
T/G box
W1-box
WUN
