# Supplementary material for: Studies on the Expression of Sesquiterpene Synthases Using Promoter-β-Glucuronidase Fusions in Transgenic Artemisia annua L
Source: PLoS One. 2013 Nov 22;8(11):e80643. doi: 10.1371/journal.pone.0080643 (PMC3838408; doi:10.1371/journal.pone.0080643)
Supplement: Figure S2 — Nucleotide sequence of the cloned ADS promoter with putative cis -acting elements shown. Putative TSS is shown in bold. Putative TATA- and CAAT-boxes are underlined. (PDF) [file pone.0080643.s002.pdf]

```
                                                               ACTTTA-motif       GT-1 box
-1875                           AGTTAATTGCACATATATGCTGAAAGTTGGCGTTGGTTTAACTTTAAACACTTCAGAAAAATCCACAAAAATTCC -1801
                                TCAATTAACGTGTATATACGACTTTCAACCGCAACCAAATTGAAATTTGTGAAGTCTTTTTAGGTGTTTTTAAGG

                        WUN       GT-1 box                                GT-1 box          E-box
-1800 AGGAAGTCACAAATAACCCAAATATTACTCCTGAAAAAGGAGAGCTCCTAAACCCCTGTGGTTTGAAAATGAAAAATCAAAAACACCATCTGAATTTTCA -1701
      TCCTTCAGTGTTTATTGGGTTTATAATGAGGACTTTTTCCTCTCGAGGATTTGGGGACACCAAACTTTTACTTTTTAGTTTTTGTGGTAGACTTAAAAGT

                   GATA-box
-1700 GACTCATAAACTCAGGATTGATAACCAATGTTCTAAAAATCAGAAAGCTTACCTCCCGACCTCTCATGACCATGTAACTTACATATTCAGAAATTATACA -1601
      CTGAGTATTTGAGTCCTAACTATTGGTTACAAGATTTTTAGTCTTTCGAATGGAGGGCTGGAGAGTACTGGTACATTGAATGTATAAGTCTTTAATATGT
                                                                       Skn-1
                                                                       RAA-motif G-box               E-box
-1600 GAAAGTACTCATGTTTAGACTCAAGAATCGCCCAAAAACACATTTCTATCATTAACTTATGACCCTTGCAACACGACCCATCTCCTGGACAGCATGCAGT -1501
      CTTTCATGAGTACAAATCTGAGTTCTTAGCGGGTTTTTGTGTAAAGATAGTAATTGAATACTGGGAACGTTGTGCTGGGTAGAGGACCTGTCGTACGTCA
                                              GATA-box      Skn-1                                      MBS
                                              box 4    WUN                              G-box
-1500 TGAGACAGAAACAAACCCTAACCCTCCTAATTCATTCATACGCCCATTATTAATTACTCTAGGGTTTTTCCAGATTTAGGTCACGTCTTAATGATCATT -1401
      ACTCTGTCTTTGTTTGGGATTGGGAGGATTAAGTAAGTATGCGGGTAATAATTAATGAGATCCCAAAAAGGTCTAAAATCCAGTGCAGAATTACTAGTAA
                                                                                GT-1 box
               TGA-element       E-box                GARE-motif AE-box CBFHV
-1400 AAATGATTTTACTAACGACTGTATTCCCCATTGATTCAAATGACAAAAACAGAAACAAGGTCGACCCATAGGATTATGGTTTCATGATTTTAGGTCTTGT -1301
      TTTACTAAAATGATTGCTGACATAAGGGGTAACTAAGTTTACTGTTTTTGTCTTTGTTCCAGCTGGGTATCCTAATACCAAAGTACTAAAATCCAGAACA
                                              Skn-1
                                 GT1-motif E-box                                          TCA-element GT-1 box
-1300 GTTTCAATTCTTATGACTTGGTACATGTTATAACAATCCACAAATGTGCTTAAACAACCATTATAGCGTGACATGAATTAAAGGAAATGAGAAGAAAAAA -1201
      CAAAGTTAAGAATACTGAACCATGTACAATATTGTTAGGTGTTTACACGAATTTGTTGGTAATATCGCACTGTACTTAATTTCCTTTACTCTTCTTTTTT
                   Skn-1                                                                   py-rich region
                                                                                      E-box
-1200 AATAGAATCGTTACCCCTTTATTTTTGTGTCGAATGAAAATGAAATCGTGAGCTCTATAGGAATTCCGCACTACACACATGGACCGAATTCAAAATGCAT -1101
      TTATCTTAGCAATGGGGAAATAAAAACACAGCTTACTTTTACTTTAGCACTCGAGATATCCTTAAGGCGTGATGTGTGTACCTGGCTTAAGTTTTACGTA
                                                                                         AU-RR core
                                                                              GATA-box
-1100 CCTAGCCTTCTTGATTGATCACCACATCGAACGGGAGGAAGAAAAGCAGCTTGGCCTCATTTTGGTGGAGATAAAAGTTCATACGCATTTTTATTTTGAT -1001
      GGATCGGAAGAACTAACTAGTGGTGTAGCTTGCCCTCCTTCTTTTCGTCGAACCGGAGTAAAACCACCTCTATTTTCAAGTATGCGTAAAAATAAAACTA
                                          Sp1
                         ACE                                  GT-1 box        E-box
-1000 GTTAGGGTTTTGAGAACAAAACCGGTTATGTATTATTTTGATGGACTTGGGCTTGGGAAAAATTTAATGGGTCAATTGGGGAGTGTTTAGAAAGGCCCAA  -901
      CAATCCCAAAACTCTTGTTTTGGCCAATACATAATAAAACTACCTGAACCCGAACCCTTTTTAAATTACCCAGTTAACCCCTCACAAATCTTTCCGGGTT
                                                 circadian                      W1-box

 -900 TGGATTTTAGTTGGGGTTAGTTTAGTCCGTGTAGCTTGTGTCGAATAATCGTGGCCCAAAAACAATTATCGGGTTTCGTTTAAATAATTATCGTAGCCCA  -801
      ACCTAAAATCAACCCCAATCAAATCAGGCACATCGAACACAGCTTATTAGCACCGGGTTTTTGTTAATAGCCCAAAGCAAATTTATTAATAGCATCGGGT
                                                                       GATA-box               GATA-box
                    box 4
 -800 AATTTAATTTACAAAAATTAATTACGGCTTATAATGACATGTTTGGTCAAAATTTTAATTATATCACATATTATACATATTACGGGTCGTAGTGAAATT  -701
      TTAAATTAAATGTTTTTAATTAATGCCGAATATTACTGTACAAACCAAGTTTTAAAATTAATATAGTGTATAATATGTATAATGCCCAGCATCACTTTAA
                                        Skn-1                    GATA-box                         WUN HSE
         MBS                   Skn-1 WUN                        E-box                              E-box
 -700 GTTTAACGGTTTACGAAATTCGGGTCATTACATACAAGCACACCCTTAAATGTTACCATTTGAGGGTTTATATAGTTATACTAGGCGCCACATATGGGCC  -601
      CAAATTGCCAAATGCTTTAAGCCCAGTAATGTATGTTCGTGTGGGAATTTACAATGGTAAACTCCCAAATATATCAATATGATCCGCGGTGTATACCCGG

                                              WUN
 -600 TTGATGGGCTTGGTGGTGTTAGACTTGCATTGTTTTACACTTTTATTACAAGCTTACACTATCCTAACACATTATCTATCATCATACAATGTCCATAGCC  -501
      AACTACCCGAACCACCACAATCTGAACGTAACAAAATGTGAAAATAATGTTCGAATGTGATAGGATTGTGTAATAGATAGTAGTATGTTACAGGTATCGG
        circadian                                                      GATA-box    GA-motif GATA-box
GATA-box ACTTTA-motif   WUN     G-box                                                               Skn-1
                                                                                                 GCN4-motif
 -500 CGATACTACTTTAATACAATTACTATCACACGTTAGAAGGTTCTTAATACAATAATGACTATCACTTCTTTTAATGCTGACACATATTGTGCTTGAGTCA  -401
      GCTATGATGAAATTATGTTAATGATAGTGTGCAATCTTCCAAGAATTATGTTATTACTGATAGTGAAGAAAATTACGACTGTGTATAACACGAACTCAGT
                           GATA-box T/G-box                      Skn-1 GATA-box          GCN4-motif
                                E-box                         GARE                    GATA-box   RAA-motif
 -400 TATTTGAAGACAATAATATTGATTACATTTGATCTTAAATTGTAGTTAGAAATAACAAGAAAACATGGATCGGTTGTTTTATAGGATATAGGACAACATG  -301
      ATAAACTTCTGTTATTATAACTAATGTAAACTAGAATTTAACATCAATCTTTATTGTTCTTTTGTACCTAGCCAACAAAATATCCTATATCCTGTTGTAC
          TC-rich repeats                                  WUN
                                                     W1-box        CGCG-box
 -300 TAGATTTAATAAATATTGTGAGTAAGACTAATATGTTTTTGAAATGTTGACCCATTCATCACGCGGGCCGTGTTGGCTAGTGTTAAAGTAATACATTGTT  -201
      ATCTAAATTATTTATAACACTCATTCTGATTATACAAAAACTTTACAACTGGGTAAGTAGTGCGCCCGGCACAACCGATCACAATTTCATTATGTAACAA
                           TCT-motif      box1 ERE WUN RAA-motif            RAA-motif  ACTTTA-motif WUN
          E-box                                                E-box
 -200 TAATAAAATTCAATTGTTTACCGGTTGTATGAAAATATTTTACCCCGTAAAAAAAAATCAATTGTTTAGGATTTAGGTCTTAGTGAGTGTGCTACTGTCA  -101
      ATTATTTTAAGTTAACAAATGGCCAACATACTTTTATAAAATGGGGCATTTTTTTTTAGTTAACAAATCCTAAATCCAGAATCACTCACACGATGACAGT
                                  E-box
                       HSE      CAAT-box                                        TATA-box
 -100 AAATATTTTACCCCATAAAAAAAATTCAATTGTTTAGGATTTAAGTCTTATTGAGTGTGCTACTGTCTTTCTATAAAATCTAGTAACTCTCCATCAAGCT    -1
      TTTATAAAATGGGGTATTTTTTTTAAGTTAACAAATCCTAAATTCAGAATAACTCACACGATGACAGAAAGATATTTTAGATCATTGAGAGGTAGTTCGA

    1 AAGAGCAACTCTAGTAAATTAAACCACATATATTCAAAGTTTTGAAAATCCATG
      TTCTCGTTGAGATCATTTAATTTGGTGTATATAAGTTTCAAAACTTTTAGGTAC
                                              box 1
```
