# Supplementary material for: Studies on the Expression of Sesquiterpene Synthases Using Promoter-β-Glucuronidase Fusions in Transgenic Artemisia annua L
Source: PLoS One. 2013 Nov 22;8(11):e80643. doi: 10.1371/journal.pone.0080643 (PMC3838408; doi:10.1371/journal.pone.0080643)
Supplement: Figure S3 — Nucleotide sequence of the cloned CPS promoter with putative cis -acting elements shown. Putative TSS is shown in bold. Putative TATA- and CAAT-boxes are underlined. (PDF) [file pone.0080643.s003.pdf]

```
                    E-box     Box I   WUN     E-box       WUN
-874 ATCGGCGTGCGGTGCATGCCTGTATCGGTGCATTGTGGTCGATTTTCATAAACGTATCAAATCTTGTCCATTCT -801
     TAGCCGCACGCCACGTACGGACATAGCCACGTAACACCAGCTAAAAGTATTTGCATAGTTTAGAACAGGTAAGA

     E-box  Box I  WUN  E-box  WUN
-800 CGTACATAGGGTTACGTAATGGTTCACATCTGATTTTTCAAATTACAAATCACATGTTTTTAGGACGTAAATTTCAATACTAAGAAAAATTCGGAAAAAA -701
     GCATGTATCCCAATGCATTACCAAGTGTAGACTAAAAAGTTTAATGTTTAGTGTACAAAAATCCTGCATTTAAAGTTATGATTCTTTTTAAGCCTTTTTT

     Skn-1
-700 AAGTTTGTCGCTCGCAAGTATATAGAAGTACCCCTCTAATGAAAGATGGTGTTATTCTCGACATAATGTGAAATCAGTCATACTTAACGATCAATAATGA -601
     TTCAAACAGCGAGCGTTCATATATCTTCATGGGGAGATTACTTTCTACCACAATAAGAGCTGTATTACACTTTAGTCAGTATGAATTGCTAGTTATTACT

     WUN  E-box
-600 TTTATGAGATTTTTAACCTTTTAAAAATTGAATTTAAGTGAGTTATTTCATTTGATTAAAAATAAGTGACTAGAATTTTCACATTTAATCTCTTATCGAT -501
     AAATACTCTAAAAATTGGAAAATTTTTAACTTAAATTCACTCAATAAAGTAAACTAATTTTTATTCACTGATCTTAAAAGTGTAAATTAGAGAATAGCTA
     GT1-motif

     T/G box
-500 TTTAATCTTTGTTGAACCAAACTTATATATAAAACCTTACTAAGTTTTGTTTTGAACTTATTAAAACAAAACGTGCGGCTCTGCCGTAACAAAGGAACAT -401
     AAATTAGAAACAACTTGGTTTGAATATATATTTTGGAATGATTCAAAACAAAACTTGAATAATTTTGTTTTGCACGCCGAGACGGCATTGTTTCCTTGTA
     RAA motif  G-box

     GATA-box  E-box
-400 CTTTATCTGATAATTAAAGGTTCCCAAATGGGTATCTTTTGTGTTCTTGCTTACACAAATATTTATATTTGTGTATTTTATACTTTTATCAGTTAATACT -301
     GAAATAGACTATTAATTTCCAAGGGTTTACCCATAGAAAACACAAGAACGAATGTGTTTATAAATATAAACACATAAAATATGAAAATAGTCAATTATGA
     as-2-box GATA-box  GATA-box MBS

     ABRE  GATA-box  RAA motif  E-box
-300 TTAAAAGTACGTGTAATCTTTTGGTAAATACTTTTATATAATTTCATGAGTTGTTAAAAATTCTTTCTTTAATTTGATAGAACAACAAATTGCCATATG -201
     AATTTTCATGCACATTAGAAAACCATTTATGAAAATATATTAAAGATCTCAACAATTTTTAAGAAAGAAATTAAACTATCTTGTTGTTTAACGGTATAC
     G-box  AAGAA-motif

     AAGAA-motif  E-box
-200 TGCTAGCTTTTCTGTTCCTGTATTATTTATCGAGTTGTATTAGAACTTGTTAGACTAAAACATGTAAAGAAATGGAGGCCAGTTAATGGCTTTTCCAAGT -101
     ACGATCGAAAAGACAAGGACATAATAAATAGCTCAACATAATCTTGAACAATCAGATTTTGTACATTTCTTTACCTCCGGTCAATTACCGAAAAGGTTCA
     MBS

     G-box
     TGACG-motif ACE  CAAT-box  TATA-box
-100 GAAAGTATTTATGACGTGGATATTTAAGGGGGTCGTTATAAAACACCAAATATTCGAACATTTTTTATGCTATAAATAGCATCATACCATCCTTAATTT -1
     CTTTCATAAATACTGCACCTATAAATTCCCCCAGCAATATTTGTGGTTTATAAGCTTTGTAAAAAATACGATATTTATCGTAGTATGGTAGGAATTAAA
     CGTCA-motif  TGA-element

     WUN  G-box  AG-motif
1    ATTCCTAAGTAGCAATTGGCTTTGAATACATAGATCCAAACTTCTCATG 49
     TAAGGATTCATCGTTAACCGAAACTTATGTATCTAGGTTTGAAGAGTAC
     Eli-box3
```
