# Supplementary material for: Studies on the Expression of Sesquiterpene Synthases Using Promoter-β-Glucuronidase Fusions in Transgenic Artemisia annua L
Source: PLoS One. 2013 Nov 22;8(11):e80643. doi: 10.1371/journal.pone.0080643 (PMC3838408; doi:10.1371/journal.pone.0080643)
Supplement: Figure S4 — Nucleotide sequence of the cloned ECS promoter with putative cis -acting elements shown. Putative TSS is shown in bold. Putative TATA- and CAAT-boxes are underlined. (PDF) [file pone.0080643.s004.pdf]

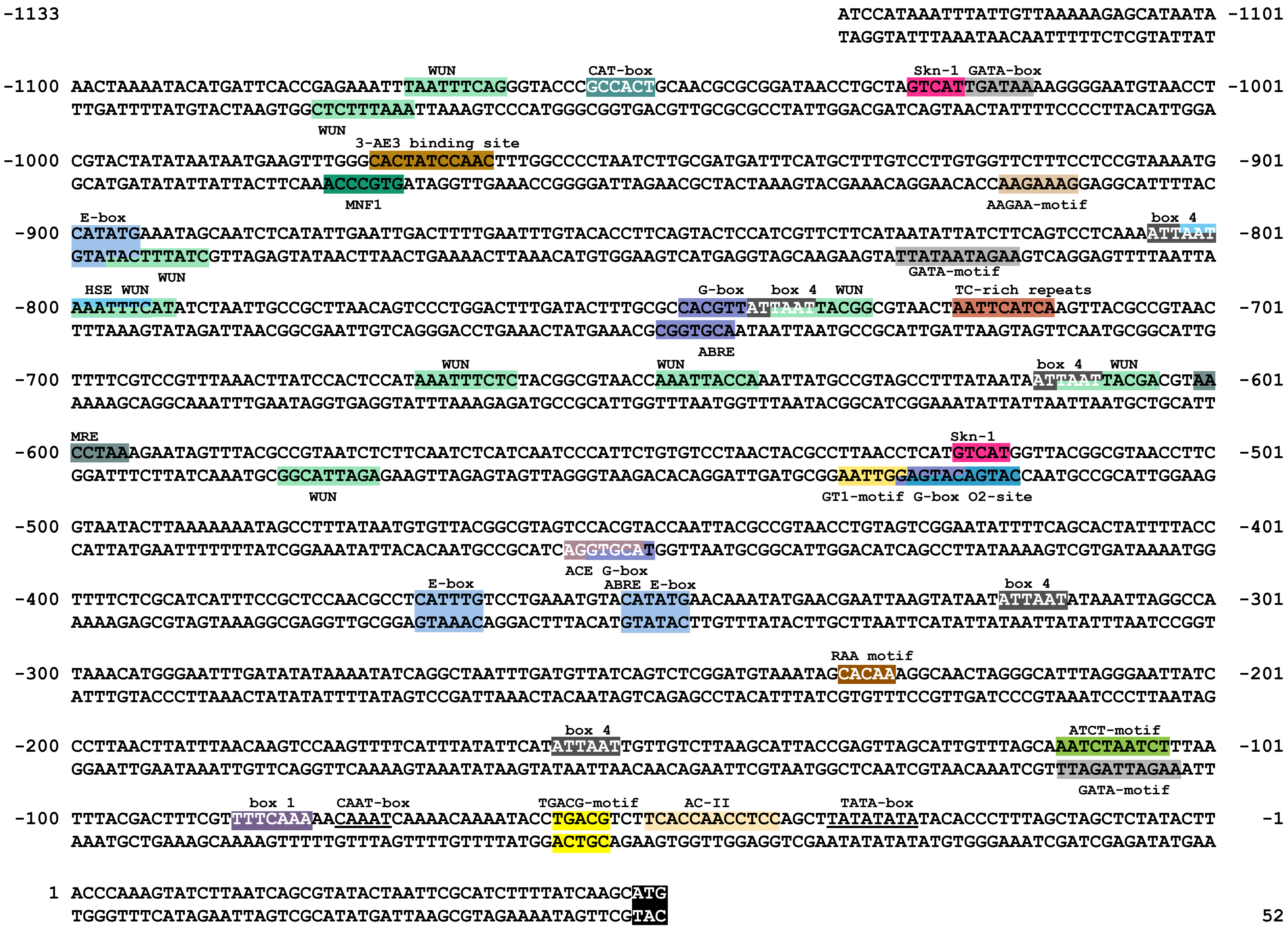
-1133 ATCCATAAATTTATTGTTAAAAAGAGCATAATA -1101
TAGGTATTTAAATAACAATTTTTCTCGTATTAT
WUN
CAT-box
Skn-1
GATA-box
-1100 AACTAAAATACATGATTCACCGAGAAATTTAATTTCAGGGTACCCGCCACTGCAACGCGCGGATAACCTGCTAGTCATTGATAAAAGGGGAATGTAACCT -1001
TTGATTTTATGTACTAAGTGGCTCTTTAAATTAAAGTCCCATGGGCGGTGACGTTGCGCGCCTATTGGACGATCAGTAACTATTTTCCCCTTACATTGGA
WUN
3-AE3 binding site
-1000 CGTACTATATAATAATGAAGTTTGGGCACTATCCAACTTTGGCCCCTAATCTTGCGATGATTTCATGCTTTGTCCTTGTGGTTCTTTCCTCCGTAAAATG -901
GCATGATATATTATTACTTCAAACCCGTGATAGGTTGAAACCGGGGATTAGAACGCTACTAAAGTACGAAACAGGAACACCAAGAAAGGAGGCATTTTAC
MNF1
AAGAA-motif
E-box
box 4
-900 CATATGAAATAGCAATCTCATATTGAATTGACTTTTGAATTTGTACACCTTCAGTACTCCATCGTTCTTCATAATATTATCTTCAGTCCTCAAAATTAAT -801
GTATACTTTATCGTTAGAGTATAACTTAACTGAAAACTTAAACATGTGGAAGTCATGAGGTAGCAAGAAGTATTATAATAGAAGTCAGGAGTTTTAATTA
WUN
GATA-motif
HSE
WUN
G-box
box 4
WUN
TC-rich repeats
-800 AAATTTCATATCTAATTGCCGCTTAACAGTCCCTGGACTTTGATACTTTGCGCCACGTTATTAATTACGGCGTAACTAATTCATCAAGTTACGCCGTAAC -701
TTTAAAGTATAGATTAACGGCGAATTGTCAGGGACCTGAAACTATGAAACGCGGTGCAATAATTAATGCCGCATTGATTAAGTAGTTCAATGCGGCATTG
ABRE
WUN
WUN
box 4
WUN
-700 TTTTCGTCCGTTTAAACTTATCCACTCCATAAATTTCTCTACGGCGTAACCAAATTACCAAATTATGCCGTAGCCTTTATAATAATTAATTACGACGTAA -601
AAAAGCAGGCAAATTTGAATAGGTGAGGTATTTAAAGAGATGCCGCATTGGTTTAATGGTTTAATACGGCATCGGAAATATTATTAATTAATGCTGCATT
MRE
Skn-1
-600 CCTAAAGAATAGTTTACGCCGTAATCTCTTCAATCTCATCAATCCCATTCTGTGTCCTAACTACGCCTTAACCTCATGTCATGGTTACGGCGTAACCTTC -501
GGATTTCTTATCAAATGCGGCATTAGAGAAGTTAGAGTAGTTAGGGTAAGACACAGGATTGATGCGGAATTGGAGTACAGTACCAATGCCGCATTGGAAG
WUN
GT1-motif
G-box
O2-site
-500 GTAATACTTAAAAAAATAGCCTTTATAATGTGTTACGGCGTAGTCCACGTACCAATTACGCCGTAACCTGTAGTCGGAATATTTTCAGCACTATTTTACC -401
CATTATGAATTTTTTTATCGGAAATATTACACAATGCCGCATCAGGTGCATGGTTAATGCGGCATTGGACATCAGCCTTATAAAAGTCGTGATAAAATGG
ACE
G-box
E-box
ABRE
E-box
box 4
-400 TTTTCTCGCATCATTTCCGCTCCAACGCCTCATTTGTCCTGAAATGTACATATGAACAAATATGAACGAATTAAGTATAATATTAATATAAATTAGGCCA -301
AAAAGAGCGTAGTAAAGGCGAGGTTGCGGAGTAAACAGGACTTTACATGTATACTTGTTTATACTTGCTTAATTCATATTATAATTATATTTAATCCGGT
RAA motif
-300 TAAACATGGGAATTTGATATATAAAATATCAGGCTAATTTGATGTTATCAGTCTCGGATGTAAATAGCACAAAGGCAACTAGGGCATTTAGGGAATTATC -201
ATTTGTACCCTTAAACTATATATTTTATAGTCCGATTAAACTACAATAGTCAGAGCCTACATTTATCGTGTTTCCGTTGATCCCGTAAATCCCTTAATAG
box 4
ATCT-motif
-200 CCTTAACTTATTTAACAAGTCCAAGTTTTCATTTATATTCATATTAATTGTTGTCTTAAGCATTACCGAGTTAGCATTGTTTAGCAAATCTAATCTTTAA -101
GGAATTGAATAAATTGTTCAGGTTCAAAAGTAAATATAAGTATAATTAACAACAGAATTCGTAATGGCTCAATCGTAACAAATCGTTTAGATTAGAAATT
GATA-motif
box 1
CAAT-box
TGACG-motif
AC-II
TATA-box
-100 TTTACGACTTTCGTTTTCAAAAACAAATCAAAACAAAATACCTGACGTCTTCACCAACCTCCAGCTTATATATATACACCCTTTAGCTAGCTCTATACTT -1
AAATGCTGAAAGCAAAAGTTTTTGTTTAGTTTTGTTTTATGGACTGCAGAAGTGGTTGGAGGTCGAATATATATATGTGGGAAATCGATCGAGATATGAA
1 ACCCAAAGTATCTTAATCAGCGTATACTAATTCGCATCTTTTATCAAGCATG
TGGGTTTCATAGAATTAGTCGCATATGATTAAGCGTAGAAAATAGTTCGTAC 52
