# Supplementary material for: Studies on the Expression of Sesquiterpene Synthases Using Promoter-β-Glucuronidase Fusions in Transgenic Artemisia annua L
Source: PLoS One. 2013 Nov 22;8(11):e80643. doi: 10.1371/journal.pone.0080643 (PMC3838408; doi:10.1371/journal.pone.0080643)
Supplement: Figure S5 — Nucleotide sequence of the cloned FS promoter with putative cis -acting elements shown. Putative TSS is shown in bold. Putative TATA- and CAAT-boxes are underlined. (PDF) [file pone.0080643.s005.pdf]

```
                                              ARE          ARE                         ARE
-1482      ATCACTCAAGGATGGATGTATTTCGTTGGTTTTTCATGGTTTTTTTTTGTTTGTTTGTTTTCATGGTTTTTTTTTGTTTGTT -1401
           TAGTGAGTTCCTACCTACATAAAGCAACCAAAAAGTACCAAAAAAAAACAAACAAACAAAAGTACCAAAAAAAAACAAACAA

                     GATA-box                GATA-box                                                  Box I
-1400 TGTTTGTTTGTTTTTTTAGATAGATTTTATTTTCAGATATTTTTTTGCATAGTTTTTTTTTTTTTTTTTTTTTTTTTTTTTTTTTAGATTTACTTTCAA -1301
      ACAAACAAACAAAAAAATCTATCTAAAATAAAAGTCTATAAAAAAAACGTATCAAAAAAAAAAAAAAAAAAAAAAAAAAAAAAAAAATCTAAATGAAAGTT
                                          HSE
                                   Box I
-1300 ATTTGAATTTTTTTTAGGTAGATTTTATTTTCAAATCTTTTTTTTATAGTTTTTTTTTTTTTTTTTTTTTTTTTTTTTTTTTGAAGATTATACTTTCAATT -1201
      TAAACTTAAAAAAAATCCATCTAAAATAAAAGTTTAGAAAAAAAATATCAAAAAAAAAAAAAAAAAAAAAAAAAAAAAAAAAACTTCTAATATGAAAGTTAA
           HSE
               GATA-box         Box I            GATA-box
-1200 TTGATTTTTTTTAGATAGATTTTATTTTCAAATCTTTTTTTTTGATAGATTTTGTGTGTGTGTTTTTTTTTTTAGATTTTACTTTTAAATTTGAATTT -1101
      AACTAAAAAAAATCTATCTAAAATAAAAGTTTAGAAAAAAAAAACTATCTAAAACACACACACACAAAAAAAAAAATCTAAAATGAAAATTTAAACTTAAA
                                                                                                   HSE
         GATA-box         Box I                                         GCN4 motif
-1100 TTTTAGATAGATTTTATTTTCAAATCTTTTTTTTTTTTTTTTTTTTTTTTACCAAATCTGATTTTGTGTCAACCAAACACGATTTCGAATTTTTAACCCCCA -1001
      AAAATCTATCTAAAATAAAAGTTTAGAAAAAAAAAAAAAAAAAAAAAAAATGGTTTAGACTAAAACACAGTTGGTTTGTGCTAAAGCTTAAAAATTGGGGGT
                                                                                                GT1-motif
                           WUN               G-box
-1000 CCATTTAAACACCTCAATGTTAAAAATTACCCCGGTTTCTAAATCCACGTTTTTTAAATTTGTGAAACAAACGCCCCCTTATATGTAGAATTTTGGAACT  -901
      GGTAAATTTGTGGAGTTACAATTTTTAATGGGGCCAAAGATTTAGGTGCAAAAAATTTAAACACTTTGTTTGCGGGGGAATATACATCTTAAAACCTTGA
                                               ACE  T/G-box
          Box 4    RAA-motif  WUN  chs-CMA1a                          RAA-motif                               WUN
 -900 TATAATGATTAATTTTCAACATAAAATTACTTAAGTATAAATATAGAATTTTAGTATCACAACAGAACTAACAAAAGGTATTTTATTAGCACCTTAATTG  -801
      ATATTACTAATTAAAAGTTGTATTTTAATGAATTCATATTTATATCTTAAAATCATAGTGTTGTCTTGATTGTTTTCCATAAAATAATCGTGGAATTAAC
                                                          GATA-box  GARE          P-box
                  WUN        G-box     GATA-box      Box-4                                 GAG-motif
 -800 CTTACATATATTATTTCATGCACACGTCCAGTCAGATAATATGTATATTAATAATTATATTCCTGTAAGAAAATCAAGAGAGTAGTCTATTTTTTTTTAT  -701
      GAATGTATATAATAAAGTACGTGTGCAGGTCAGTCTATTATACATATAATTATTAATATAAGGACATTCTTTTAGTTCTCTCATCAGATAAAAAAAAATA
                                                                   TCT-motif TC-rich repeats
                                    GAG-motif                  GATA-box   RAA-motif
 -700 TATTATTATTATTTTTTTTAACGGCGGTTTAAGAGAGTAGTCTATTTAGTGATGGATACATGCACAACAAGGCTAATTTATTTGATTTTAATAGTTACAT  -601
      ATAATAATAATAAAAAAAATTGCCGCCAAATTCTCTCATCAGATAAATCACTACCTATGTACGTGTTGTTCCGATTAAATAAACTAAAATTATCAATGTA

      Box 4       Skn-1                                            Skn-1
 -600 TAATCTCAGTATGTCATTATCTCTAGTACTTCTTCATCTCCGTTTCCATCAGTTTAAAGAATGTCATAATAATAGAACAGTCTTTATATATTTAAAAAAA  -501
      ATTAGAGTCATACAGTAATAGAGATCATGAAGAAGTAGAGGCAAAGGTAGTCAAATTTCTTACAGTATTATTATCTTGTCAGAAATATATAAATTTTTTT
                      GATA-box        GAG-motif
                                                                                                RY-element
 -500 CATATTTAACCAAAAATTAAAGTAAACCTTATATATAATCACTATTTTAGTAAGCTAAGTGACAACTTTTACATTAACTACGACAACGCATGCATGTTCG  -401
      GTATAAATTGGTTTTTAATTTCATTTGGAATATATATTAGTGATAAAATCATTCGATTCACTGTTGAAAATGTAATTGATGCTGTTGCGTACGTACAAGC
           GT1-motif     ATTTCA-motif
                                                                          GATA-box
 -400 TTGGTTATTTTGTATACTGTTATATATTCAAGCAGGACGTGTAACATTTTCTCAGATTTATCTCCGATACTTAAATCTTTACCGGTAACTAACATAAGTT  -301
      AACCAATAAAACATATGACAATATATAAGTTCGTCCTGCACATTGTAAAAGAGTCTAAATAGAGGCTATGAATTTAGAAATGGCCATTGATTGTATTCAA
         L-box                            G-box              GATA-box
                                                                            TCT-motif
 -300 TTCTTTAATGCATCTTGCTTGGATGATGGTAGAAAAACTAGCGCAATTCTCGGCTAGAGTAGTCTTACGTACATGCCTATAAAAACACTAATGACATTAA  -201
      AAGAAATTACGTAGAACGAACCTACTACCATCTTTTTGATCGCGTTAAGAGCCGATCTCATCAGAATGCATGTACGGATATTTTTGTGATTACTGTAATT
                                                                                                  Skn-1
        RAA-motif                             WUN
 -200 CACAACAAAAAATAACTAACCCTATTTATTTACGTAGTACGTAATTTCCTTAACTAGTCTTCCTTATTTTGGAATAAATTAAAATTGTGACAGTTAGAAA  -101
      GTGTTGTTTTTTATTGATTGGGATAAATAAATGCATCATGCATTAAAGGAATTGATCAGAAGGAATAAAACCTTATTTAATTTTAACACTGTCAATCTTT
                                                                GA-motif       WUN                MBS TC-rich repeats
                                CAAT-box         E-box                                TATA-box TC-rich repeats
 -100 ATGTTTGTACTTGTTTTGTCCTAGCTGTCAATTATGAGATGCACATGAACATGTTCCTCTTGCATTGCCTATATATTTGCTTCACTTTGATTTCATTTTC    -1
      TACAAACATGAACAAAACAGGATCGACAGTTAATACTCTACGTGTACTTGTACAAGGAGAACGTAACGGATATATAAACGAAGTGAAACTAAAGTAAAAG

    1 AATATCAAACTCGCAAGATTTTGAGATG                                                                            28
      TTATAGTTTGAGCGTTCTAAAACTCTAC
       GATA-box
```
